# Exact parameter identification in PET pharmacokinetic modeling using the irreversible two tissue compartment model^*^ *This work was partly funded by the NIH Grants P41-EB017183 and R01-EB031199-02.


**DOI:** 10.1088/1361-6560/ad539e

**Published:** 2024-07-30

**Authors:** Martin Holler, Erion Morina, Georg Schramm

**Affiliations:** 1 Department of Mathematics and Scientific Computing, University of Graz, Graz, Austria; 2 Radiological Sciences Laboratory, Stanford University, Stanford, CA, United States of America; 3 Department of Imaging and Pathology, KU Leuven, Leuven, Belgium

**Keywords:** quantitative PET imaging, two-tissue compartment model, exact reconstruction, Tikhonov regularization, iteratively regularized Gauss Newton method

## Abstract

*Objective.* In quantitative dynamic positron emission tomography (PET), time series of images, reflecting the tissue response to the arterial tracer supply, are reconstructed. This response is described by kinetic parameters, which are commonly determined on basis of the tracer concentration in tissue and the arterial input function. In clinical routine the latter is estimated by arterial blood sampling and analysis, which is a challenging process and thus, attempted to be derived directly from reconstructed PET images. However, a mathematical analysis about the necessity of measurements of the common arterial whole blood activity concentration, and the concentration of free non-metabolized tracer in the arterial plasma, for a successful kinetic parameter identification does not exist. Here we aim to address this problem mathematically. *Approach.* We consider the identification problem in simultaneous pharmacokinetic modeling of multiple regions of interests of dynamic PET data using the irreversible two-tissue compartment model analytically. In addition to this consideration, the situation of noisy measurements is addressed using Tikhonov regularization. Furthermore, numerical simulations with a regularization approach are carried out to illustrate the analytical results in a synthetic application example. *Main results.* We provide mathematical proofs showing that, under reasonable assumptions, all metabolic tissue parameters can be uniquely identified without requiring additional blood samples to measure the arterial input function. A connection to noisy measurement data is made via a consistency result, showing that exact reconstruction of the ground-truth tissue parameters is stably maintained in the vanishing noise limit. Furthermore, our numerical experiments suggest that an approximate reconstruction of kinetic parameters according to our analytic results is also possible in practice for moderate noise levels. *Significance.* The analytical result, which holds in the idealized, noiseless scenario, suggests that for irreversible tracers, fully quantitative dynamic PET imaging is in principle possible without costly arterial blood sampling and metabolite analysis.

## 1. Introduction

Positron emission tomography (PET) is a non-invasive clinical technique that images the four dimensional spatio temporal distribution of a radio tracer *in*-*vivo*. In quantitative dynamic PET imaging, several 3D PET images are acquired at different time points after tracer injection and reconstructed. As a response to the supply of tracer via the arteries and capillaries, the tracer is exchanged with tissues. This exchange can include reversible and/or irreversible binding and eventually metabolization of the tracer.

Using the right tracer, the time series of reconstructed PET images reflecting the tissue response, a measurement of the arterial tracer input, and a dedicated pharmacokinetic model, it is possible to generate images reflecting certain physiological parameters. Depending on the tracer, these parametric images can reflect e.g. blood flow, blood volume, glucose metabolism or neuro receptor dynamics.

**Figure 1.**
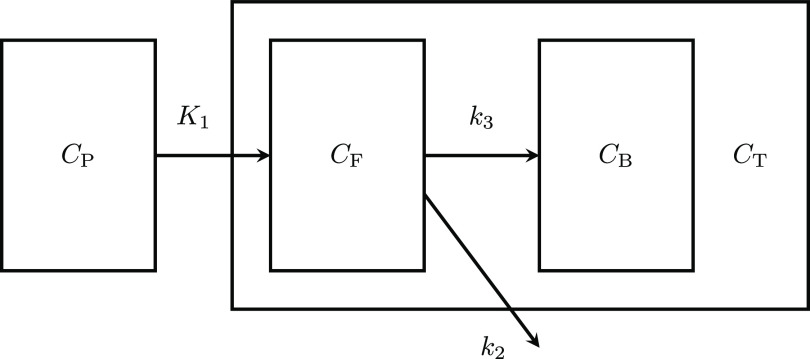
Irreversible two tissue compartment model. The boxes around $C_{\textrm{P}}, C_{\textrm{B}}, C_{\textrm{F}}$ and *$C_{\textrm{T}}$* represent the respective concentrations and the arrows between them the directional exchange with the respective rate depicted above or below the corresponding arrow. The box around the *$C_{\textrm{B}}$* and *$C_{\textrm{F}}$* indicates the extra-vascular measurements associated with *$C_{\textrm{T}}$*.

Pharmacokinetic modeling in PET is commonly performed using compartment models, where the compartments usually reflect tissue subspaces. Examples for such subspaces are the extra cellular spaces where the tracer is free or bound^4^ 4Note that the exact interpretation of the biological meaning of the compartments is highly non-trivial.. The dynamics between the arterial blood and tissue compartments is typically described using ordinary-differential-equation (ODE) models. For PET tracers with irreversible binding, such as [^18^F]Fluorodeoxyglucose (FDG) (Sokoloff 1978) or [^11^C]Clorgyline (Logan *et al* 2002), the irreversible two tissue compartment model can be used to describe the tracer dynamics, see figure 1 for a scheme of this model.

The identification of the kinetic parameters describing the tissue response to the arterial tracer supply in a given region is commonly done using the following input data:

1. The metabolite corrected plasma input curve $C_{\textrm{P}}(t)$ describing the concentration of the original non-metabolized free tracer in the arterial blood plasma that is supplied to tissue and available for exchange (and metabolism). However, a direct measurement of $C_{\textrm{P}}(t)$ is complicated. Typically, it is based on an external measurement of a time series of manual arterial blood samples taken from a patient, e.g. using a well counter. Unfortunately, this arterial whole blood tracer concentration $C_{\textrm{WB}}(t)$ obtained from well counter measurements usually overestimates $C_{\textrm{P}}(t)$ since the measured activity of the blood samples also includes activity from radioactive molecules that are not available for exchange with tissue because of (i) parts of the radio tracer being bound to plasma proteins or membranes of blood cells and (ii) activity originating from metabolized tracer molecules that were transfered back from tissue into blood. Measuring the contributions of the latter two effects, summarized in the ratio $f(t) = C_{\textrm{P}}(t) / C_{\textrm{WB}}(t)$, requires further advanced chemical processing and analysis of the blood samples which is time consuming and expensive.
2. The tracer concentration in tissue $C_{\textrm{T}}(t)$. This quantity, which is equal to the sum of all the tracer concentrations in all extra-vascular compartments, can be obtained from the time series of reconstructed PET images—either on a region-of-interest (ROI) or at voxel level. Note that in the presence of a finite fraction blood volume ${V_{\textrm{B}}}$, the tissue time activity curves (TACs) obtained from dynamic PET images are a weighted sum of the tracer concentration in tissue and blood, i.e. $C_{\textrm{PET}}(t) = (1-{V_{\textrm{B}}})C_{\textrm{T}}(t) + {V_{\textrm{B}}} C_{\textrm{WB}}(t)$.

To avoid the necessity of arterial blood sampling, which itself is a very challenging process in clinical routine, many attempts have been made to derive the arterial input function directly from the reconstructed dynamic PET images itself (also called ‘image-based arterial input function’) by analyzing the tracer concentration in regions of interest of the PET images containing arterial blood, such as the left ventricle, the aorta, or the carotic arteries. Note, however, that by using any image-based approach for the estimation of the arterial input function, the contributions of tracer bound to plasma proteins and metabolized tracers cannot be determined. In other words, any image-based approach can only estimate $C_{\textrm{WB}}(t)$ instead of $C_{\textrm{P}}(t)$.

Motivated by this problem, we consider the question whether the identification of kinetic parameters for tracers with irreversible binding is possible without measuring the function *$f(t)$* and/or the arterial whole blood tracer concentration $C_{\textrm{WB}}(t)$, by simultaneously analyzing time activity curves from multiple ROIs.

In existing literature on modeling approaches for quantitative PET, see for instance Veronese *et al* (2013), Tonietto *et al* (2015), Dimitrakopoulou-Strauss *et al* (2021), van der Weijden *et al* (2023), this question has been addressed from different computational perspectives. The work Veronese *et al* (2013), for instance, accounts for a low number of measurements of the arterial concentration of non-metabolized PET tracer by using a non-linear mixed effect model for the parent plasma fraction, i.e. the parameters defining the parent plasma fraction are modeled as being partially patient-specific and partially the same for a population sample. Moreover, the general idea of jointly modeling the tissue response in different anatomical regions to obtain unknown common parameters or to reduce the variance in the estimated region-dependent parameters has been proposed in Raylman *et al* (1994), Huesman and Coxson (1997), Todd Ogden *et al* (2015), Chen *et al* (2019), Matheson and Todd Ogden (2022, 2023).

For the specific case of simultaneous estimation of kinetic parameters and the input function of [^18^F]-FDG data using the irreversible two tissue compartment model, Roccia *et al* (2019) applied the method of non-invasive simultaneous estimation (nSIME) of multiple TACs from different ROIs assuming a common arterial input function. nSIME (Mikhno *et al* 2015) is an extension of SIME (Feng *et al* 1997, Wong *et al* 2001, Todd Ogden *et al* 2010) aiming to replace the single blood sample needed by SIME with a prediction based on a machine learning model applied to electronic health record data of the patient. Compared to the gold standard of kinetic modeling with arterial blood sampling, the best nSIME method showed high correlation (*r* = 0.83) and low bias when estimating the regional cerebral metabolic rate of glucose and performed better than method using a population-based input function (Takikawa *et al* 1993). Recently, Liang *et al* (2023) proposed a method combining deep learning and kinetic modeling to directly estimate all kinetic parameters and the fractional blood volume for 30 min of dynamic FDG PET data without access to the input function.

Despite these and many more computational approaches for parameter identification in pharmacokinetic modeling using the irreversible two tissue compartment model, a mathematical analysis (in the sense of mathematical proofs) about the sufficiency of measurements of $C_{\textrm{WB}}(t)$ and/or *$f(t)$* for successful parameter identification, even in an idealized, noiseless scenario, does not exist. In addition to that, even in the presence of an arbitrary number of such measurements, we are not aware of mathematical proofs that guarantee unique recovery of the tissue parameters in this specific ODE-based compartment model. To the best of our knowledge, these questions can also not be answered as special case of classical identifiability results such as Banks and Kunisch (1989, theorem 4.1) (one reason being lack of regularity, i.e. the first Gâteaux variation of the parameter-to-state map is neither bounded nor coercive).

The aim to this work is hence to answer these questions mathematically. Using a polyexponential parametrization of the arterial plasma concentration, which is frequently used in practice, we prove the following:

Theorem 1 **(main paper result, informal version).**

Let $(K_1^i,k_2^i,k_3^i)$ be the kinetic parameters of different anatomical regions $i = 1,\ldots,n$ of the irreversible two tissue compartment model, let $T$ be the number of time-points where PET measurements of the tissue tracer concentration $C_{\textrm{T}}(t)$ are available, and let *$p$* be the degree of the polyexponential parametrization of the arterial plasma concentration.

• If $T\unicode{x2A7E}2(p+3)$, and under some non-restrictive technical conditions as stated in theorem 15, the parameters $k_2^i,k_3^i$ for $i = 1,\ldots,n$ can be identified uniquely already from the available image-based measurements of the tracer concentration in the different tissues without the need for $C_{\textrm{WB}}(t)$ and *$f(t)$*.
• Further, the $K_1^i$ can also be identified already from these measurements up to a constant that is the same for all regions $i$.
• In addition, the parameters $K_1^i$ can be identified exactly if a sufficient number of measurements of the total arterial tracer concentration *$C_{\textrm{WB}}$* is available, without the need for *$f(t)$*.

A precise statements and proof of these results can be found in theorem 15 below. Regarding practical application, this result means that the relevant tissue parameters $(K_1^i,k_2^i,k_3^i)$, $i = 1,\ldots,n$, can, in principle, be uniquely recovered (up to a global constant for the $K^i_1$) from image based measurement of the tracer concentration in the different tissue types, provided that image-based measurements of sufficiently high quality and at sufficiently many time-points (e.g. $T\unicode{x2A7E} 14$) are available. Further, also the parameters $K_1^i$, $i = 1,\ldots,n$, can be recovered uniquely if a sufficient number of high-quality, image-based measurements of the total arterial tracer concentration is available. While these results are formulated for the case when PET images provide the tissue concentration *$C_{\textrm{T}}$*, they also generalize to some extend to the case when voxel measurements provide a convex combination of tissue and blood tracer concentration, see remark 17 below for details.

Besides these unique identifiability results that consider the idealized case of a noise-free measurement, we also present analytical results for a standard Tikhonov regularization approach that addresses the situation of noisy measurements. Using classical results from regularization theory, we show that the Tikhonov regularization approach is stable w.r.t. perturbations of the data and, in the vanishing noise limit, allows to approximate the ground-truth tissue parameters. Numerical experiments further illustrate our analytic results also in an application example.

**Scope of the paper.** In section 2, we introduce the irreversible two tissue compartment model and provide basic results on explicit solutions both in the general case and in case the arterial concentration is parametrized via polyexponential functions. In section 3 we present and prove our main results on unique identifiability of parameters. In section 4 we introduce a Tikhonov regularization approach and show stability and consistency results, and in sections 5 and 6 we provide a numerical algorithm and numerical experiments for an application example. All numerical experiments of the paper can be reproduced based on the source code (Holler *et al* 2024).

## 2. The irreversible two tissue compartment model

The *irreversible two tissue compartment model* describes the interdependence of the concentration of a radio tracer in the arterial blood plasma and in the extra-vascular compartment, where the latter is further decomposed in a *free* and a *bound* compartment. Note that in the irreversible model, once the radio tracer has reached the bound compartment, it is trapped. A visualization of the model is provided in figure 1.

We denote by $C_{\textrm{P}}:[0,\infty) \rightarrow [0,\infty)$ the arterial plasma concentration of the non-metabolized PET tracer. Further, for any anatomical region $i = 1,\ldots,n$, we denote by $C^i_{\textrm{F}}:[0,\infty) \rightarrow [0,\infty)$ and $C^i_{\textrm{B}}:[0,\infty) \rightarrow [0,\infty)$ the free and the bound compartment of the tracer in region *$i$*, respectively, and by $C^i_{\textrm{T}} = C^i_{\textrm{F}} + C^i_{\textrm{B}}$ we denote the sum of the two compartments in region *$i$*. Using the *irreversible two tissue compartment model*, the interaction of these quantities is described by the following system of ODEs:

\begin{align*} \begin{cases} \frac{\mathop{}\!\mathrm{d}}{\mathop{}\!\mathrm{d} t}C^i_{\textrm{F}} = K^i_1 C_{\textrm{P}} - \left(k^i_2+k^i_3\right) C^i_{\textrm{F}}, &amp; t&gt;0 \\ \frac{\mathop{}\!\mathrm{d}}{\mathop{}\!\mathrm{d} t}C^i_{\textrm{B}} = k^i_3 C^i_{\textrm{F}}, &amp; t&gt;0 \\ C^i_{\textrm{F}}\left(0\right) = 0, ~ C^i_{\textrm{B}}\left(0\right) = 0 \end{cases}.\end{align*}

Here, the parameters $K_1^i$, $k_2^i$ and $k_3^i$ are the tracer kinetic parameters that define the interaction of the different compartments in region *$i$*.

Our goal is to identify the parameters $K_1^i$, $k_2^i$ and $k_3^i$ for each $i = 1,\ldots,n$. For this, we can use measurements of the $C_{\textrm{T}}^i(t_l)$ at different time-points $t_1,\ldots,t_T$ obtained from reconstructed PET images at different time points after tracer injection. Further, the parameter identification typically relies on additional measurements related to *$C_{\textrm{P}}$*. Here, the standard procedure is to take arterial blood samples and to measure the total activity concentration of the arterial blood samples, given as $C_{\textrm{WB}}:[0,\infty) \rightarrow [0,\infty)$. The relation of the total concentration *$C_{\textrm{WB}}$* to the arterial plasma concentration *$C_{\textrm{P}}$* of non-metabolized tracer is described via an unknown function $f:[0,\infty) \rightarrow [0,1]$ with $f(0) = 1$ as

\begin{align*} C_{\textrm{P}}\left(t\right) = f\left(t\right)C_{\textrm{WB}}\left(t\right).\end{align*}

As described above, to obtain *$f(t)$* and thus $C_{\textrm{P}}(t)$, a time-consuming and costly plasma separation and metabolite analysis of the blood samples has to be performed.

In order to realize the parameter identification for the ODE model (S) in practice, the involved functionals need to be discretized, e.g. via a suitable parametrization. For the arterial concentration *$C_{\textrm{P}}$*, it is standard to use a parametrization via polyexponential functions as defined in the following.

Definition 2 **(polyexponential functions).**

We call a function *$g$* polyexponential of degree $p\in\mathbb{N}$ if there exist $\lambda_i, \mu_i\in\mathbb{R}$ for $1\unicode{x2A7D} i\unicode{x2A7D} p$ where the $\left(\mu_i\right)_{i = 1}^p$ are pairwise distinct and $\lambda_i\neq 0$ for all *$i$* such that

\begin{align*} g\left(t\right) = \sum_{i = 1}^p\lambda_i e^{\mu_i t}.\end{align*}

We write $\deg\left(g\right) = p$ and call the zero-function polyexponential of degree zero. By $\mathcal{P}_p$ we denote the set of polyexponential functions of degree less or equal to *$p$*, and by $\mathcal{P} = \cup_{p = 0}^\infty \mathcal{P}_p$ the set of polyexponential functions (of any degree).

Remark 3. It obviously holds that $\mathcal{P}$ is a vector space, and even a subalgebra of $\mathcal{C}^\infty\left(\mathbb{R}\right)$. It is also worth noting that, as direct consequence of the Stone-Weierstrass Theorem, polyexponential functions are dense in the set of continuous functions on compact domains. Thus, they are a reasonable approximation class also from the analytic perspective.

Modeling *$C_{\textrm{P}}$* as polyexponential function already defines a parametrization of the resulting solutions of the ODE system (S). As we will see in lemma 6 below, the following notion of generalized polyexponential functions is the appropriate notion to describe such solutions.

Definition 4 **(generalized polyexponential class).**

We call a function *$g$* generalized polyexponential if it is of the form

\begin{align*} g\left(t\right) = P_1\left(t\right)e^{\mu_1 t}+\dots+P_l\left(t\right)e^{\mu_l t},\end{align*}

where the $P_1, \dots, P_l\not\equiv 0$ are polynomials of degree $m_1-1, \dots, m_l-1$, respectively, and the *$\mu_j$* are pairwise distinct constants. We denote the class of such generalized polyexponential functions with polynomials of degree at most $m_1-1,\ldots,m_l-1$ by

\begin{align*} \mathcal{P}\left[m_1, \dots, m_l\right].\end{align*}

We define the degree of *$g$* by

\begin{align*} \deg\left(g\right) = m_1+\dots+m_l.\end{align*}

In case $m_1 = \dots = m_l = 1$ we write $\mathcal{P}_{\deg\left(g\right)}$ for the resulting polyexponential class.

The next two results, which follow from standard ODE theory, provide explicitly the solutions $(C_{\textrm{F}},C_{\textrm{B}})$ of the ODE system (S), once in the general case and once in case *$C_{\textrm{P}}$* is modeled as polyexponential function.

Lemma 5. Let $C_{\mathrm{P}}:[0,\infty) \rightarrow [0,\infty)$ be continuous, and let the parameters $K_1^i$, $k_2^i$ and $k_3^i$ be fixed for $i = 1,\ldots,n$ such that $k_2^i + k_3^i \neq 0$. Then, for each $i = 1,\ldots,n$, the ODE system (S) admits a unique solution $(C_{\mathrm{F}}^i,C_{\textrm{B}}^i)$ that is defined on all of $[0,\infty)$, and such that $C_{\mathrm{T}}^i = C_{\mathrm{F}}^i + C_{\mathrm{B}}^i$ is given as

\begin{equation*} C_{\mathrm{T}}^i(t) = \frac{K_1^ik_2^i}{k_2^i+k_3^i}e^{-\left(k_2^i+k_3^i\right)t}\int_0^t e^{\left(k_2^i+k_3^i\right)s} C_{\mathrm{P}}\left(s\right)\mathop{}\!\mathrm{d} s+\frac{K_1^ik_3^i}{k_2^i+k_3^i}\int_0^t C_{\mathrm{P}}\left(s\right)\mathop{}\!\mathrm{d} s.\end{equation*}

Proof. Fix $i \in \{1,\ldots,n\}$. From the equation for $C_{\textrm{F}}^i$ in (S) it immediately follows that

\begin{equation*} C_{\textrm{F}}^i\left(t\right) = K_1^i e^{-\left(k_2^i+k_3^i\right)t}\int_0^t e^{\left(k_2^i+k_3^i\right)s}C_{\textrm{P}}\left(s\right)\mathop{}\!\mathrm{d} s.\end{equation*}

This in turn implies that

\begin{align*} C_{\textrm{B}}^i\left(t\right) = -\frac{K_1^i k_3^i}{k_2^i+k_3^i}e^{-\left(k_2^i+k_3^i\right)t}\int_0^t e^{\left(k_2^i+k_3^i\right)s}C_{\textrm{P}}\left(s\right)\mathop{}\!\mathrm{d} s+\frac{K_1^i k_3^i}{k_2^i+k_3^i}\int_0^t C_{\textrm{P}}\left(s\right)\mathop{}\!\mathrm{d} s\end{align*}

and, consequently, that

\begin{align*} C_{\textrm{T}}^i\left(t\right) = \frac{K_1^ik_2^i}{k_2^i+k_3^i}e^{-\left(k_2^i+k_3^i\right)t}\int_0^t e^{\left(k_2^i+k_3^i\right)s} C_{\textrm{P}}\left(s\right)\mathop{}\!\mathrm{d} s+\frac{K_1^ik_3^i}{k_2^i+k_3^i}\int_0^t C_{\textrm{P}}\left(s\right)\mathop{}\!\mathrm{d} s\end{align*}

as claimed. □

Lemma 6. If $C_{\textrm{P}}\in\mathcal{P}_p$ is given as

\begin{align*} C_{\textrm{P}}\left(t\right) = \sum_{j = 1}^p \lambda_j e^{\mu_j t},\end{align*}

then, for $i \in \{1,\ldots,n\}$, $C_{\textrm{T}}^i$ of lemma 5 is given as

\begin{align*} C_{\textrm{T}}^i\left(t\right) &amp;= \frac{K_1^i}{k_2^i+k_3^i}\sum_{j = 1}^p\left( \frac{k_3^i}{\mu_j}\mathbf{1}_{\left\{\mu_j \neq 0\right\}}+\frac{k_2^i}{k_2^i+k_3^i+\mu_j}\mathbf{1}_{\left\{k_2^i+k_3^i+\mu_j\neq 0\right\}}\right)\lambda_je^{\mu_jt} \nonumber\\&amp;\quad -\left[\frac{K_1^ik_2^i}{k_2^i+k_3^i}\sum_{\substack{j = 1 \\ k_2^i+k_3^i+\mu_j\neq 0}}^p\frac{\lambda_j}{k_2^i+k_3^i+\mu_j}\right]e^{-\left(k_2^i+k_3^i\right)t} -\frac{K_1^ik_3^i}{k_2^i+k_3^i}\sum_{\substack{j = 1 \\ \mu_j\neq 0}}^p \frac{\lambda_j}{\mu_j} \nonumber\\ &amp;\quad +\left[\frac{K_1^ik_2^i}{k_2^i+k_3^i}\sum_{\substack{j = 1 \\ k_2^i+k_3^i+\mu_j = 0}}^p\lambda_j\right]te^{-\left(k_2^i+k_3^i\right)t} + \frac{K_1^ik_3^i}{k_2^i+k_3^i}\sum_{\substack{j = 1 \\ \mu_j = 0}}^p \lambda_j t.\end{align*}

Proof. This follows immediately by inserting the representation of *$C_{\textrm{P}}$* in (1). □

Remark 7 **(sign of exponents *µ*_*j*_).**

Note that for the ground truth arterial concentration *$C_{\textrm{P}}$*, we will always have $\mu_j < 0$ (in particular $\mu_j \neq 0$) for all $j = 1,\ldots,p$, since otherwise this would imply the unphysiological situation that $C_{\textrm{P}}(t) \rightarrow c \neq 0$ for $t \rightarrow \infty$.

## 3. Unique identifiability

In view of lemma 6 from the previous section, it is clear that the question of unique identifiability of the parameters $K_1^i$, $k_2^i$ and $k_3^i$ from measurements of $C_{\textrm{T}}^i(t_l)$ at time points $t_1,\ldots,t_T$ is related to the question of uniqueness of interpolations with generalized polyexponential functions. A first, existing result in that direction is as follows.

Lemma 8 **(roots of generalized polyexponential functions).**

Let $P_1, \dots, P_l$ be polynomials of degree $m_1-1, \dots, m_l-1$ such that at least one of them is not identically zero, and let the constants $\mu_1, \dots, \mu_l$ be pairwise distinct. Then the function

\begin{align*} g\left(t\right) = P_1\left(t\right)e^{\mu_1 t}+\dots+P_l\left(t\right)e^{\mu_l t}\end{align*}

admits at most $m_1+\dots+m_l-1$ real roots.

Proof. See Polya and Szegö (1971, exercise 75 (p 48)). □

As a consequence of the previous proposition, we now obtain the following unique interpolation result for generalized polyexponential functions.

Lemma 9 **(unique interpolation).**

Let $m_1,\dots, m_p, T\in \mathbb{N}$ be such that

\begin{align*} 2\left(m_1+\dots+m_p\right)\unicode{x2A7D} T.\end{align*}

Then, for any choice of tuples $\left(t_i, s_i\right)\in \mathbb{R}^2$, $i = 1,\ldots,T$, with $t_1 < \dots < t_T$, there exists at most one generalized polyexponential function $h\in\mathcal{P}\left[m_1, \dots, m_p\right]$ such that

\begin{equation*} h\left(t_l\right) = s_l\end{equation*}

for $l = 1,\ldots,T$, i.e. in case

\begin{align*} h\left(t\right) = \sum_{j = 1}^p P_j\left(t\right)e^{\mu_j t} \quad \textrm{and}\quad \tilde h\left(t\right) = \sum_{j = 1}^p \tilde P_j\left(t\right)e^{\tilde \mu_j t}\end{align*}

are two generalized polyexponential functions with $h,\tilde{h}\in\mathcal{P}\left[m_1, \dots, m_p\right]$ fulfilling the interpolation condition (4), then, up to re-indexing,

\begin{align*} P_j \equiv \tilde{P_j}\end{align*}

for all *$j$* and $\mu_j = \tilde{\mu}_j$ for all *$j$* where $P_j \not\equiv 0$.

Proof. Let both $h, \tilde{h}\in\mathcal{P}\left[m_1, \dots, m_p\right]$ fulfill the interpolation condition (4), and, w.l.o.g. assume that $P_j \not \equiv 0$ and $\tilde{P}_j \not \equiv 0$ for all $j$. Then, $h - \tilde{h} \in \mathcal{P}\left[m_1, \dots, m_p,m_1,\ldots,m_p\right]$ and $(h-\tilde{h})(t_l) = 0$ for $l = 1,\ldots,T$. Lemma 8 then implies that all polynomials appearing in $h-\tilde{h}$ in a representation as in definition 4 are identically zero. This implies that the $(P_j)_{j = 1}^p$ and $(\tilde P_j)_{j = 1}^p$ coincide up to re-indexing and, likewise, that the corresponding coefficient $(\mu_j)_{j = 1}^p$ and $(\tilde \mu_j)_{j = 1}^p$ where the corresponding polynomials are non-zero coincide as well. □

Based on this result, we now address the question of uniquely identifying the parameters of the ODE system (S) from time-discrete measurements $C_{\textrm{T}}^i(t_1), \ldots,C_{\textrm{T}}^i(t_T)$ with $i = 1,\ldots,n$ and measurements $C_{\textrm{WB}}(s_1),\ldots,C_{\textrm{WB}}(s_q)$. For this, we first introduce the following notation.

Definition 10 **(parameter configuration).**

We call the parameters $p,n\in \mathbb{N}$, $((\lambda_j,\mu_j))_{j = 1}^p \in \mathbb{R}^{2 \times p} $, $((K_1^i,k_2^i,k_3^i))_{i = 1}^n \in \mathbb{R}^{3 \times n}$ together with the functions $(C^i_{\textrm{T}})_{i = 1}^n$ and

\begin{align*} C_{\textrm{P}}\left(t\right) = \sum_{j = 1}^p \lambda_j e^{\mu_jt}\end{align*}

a configuration of the irreversible two tissue compartment model if $\lambda_j \neq 0$ for $j = 1,\ldots,p$, the *$\mu_j$*, $j = 1,\ldots,p$ are pairwise distinct, and, for $i = 1,\ldots,n$, $C_{\textrm{T}}^i = C_{\textrm{F}}^i + C_{\textrm{B}}^i$ with $(C_{\textrm{F}}^i,C_{\textrm{B}}^i)$ the solution of the ODE system (S) with arterial concentration *$C_{\textrm{P}}$* and parameters $K_1^i,k_2^i,k_3^i$.

Central for our unique identifiability result will be the following technical assumption on a parameter configuration $(p,n,((\lambda_j,\mu_j))_{j = 1}^p ,((K_1^i,k_2^i,k_3^i))_{i = 1}^n,(C^i_{\textrm{T}})_{i = 1}^n,C_{\textrm{P}})$

\begin{equation*} \left\{ \begin{aligned} &amp; \textrm{For any}\ j_0,\ \textrm{there are at least three regions } i_s, \, s = 1,\ldots,3, \ \textrm{where } \\ &amp; k^{i_s}_3 \ \textrm{and } k^{i_s}_2 + k^{i_s}_3 \ \textrm{are each pairwise distinct, }\mu_{j_0} + k_3^{i_s} \neq 0 \\ &amp; \textrm{and either }\mu_{j_0} + k_2^{i_s} + k_3^{i_s} = 0 \ \textrm{or } \sum_{\substack{j = 1 \\ \mu_{j} + k_2^{i_s} + k_3^{i_s} \neq 0}}^p \frac{\lambda_j}{k_2^{i_s} + k_3^{i_s} + \mu_j} \neq 0. \end{aligned} \right.\end{equation*}

As the following lemma shows, this assumption holds in case our measurement setup comprises sufficiently many regions where the parameters $k_3^i$ and $k_2^i+k_3^i$ are pairwise distinct. This is reasonable to assume in practice, and also it is a condition which is to be expected: Our unique identifiability result will require a sufficient number of different regions, and different regions with the same tissue parameter do not provide any additional information on the dynamics of the ODE model.

Lemma 11. Assume that there are at least $p+3$ regions $i_{1},\ldots,i_{p+3}$, with $p\unicode{x2A7E} 1$, where each the $k^{i_s}_3$ and the $k^{i_s}_2 + k^{i_s}_3$ are pairwise distinct for $s = 1,\ldots,p+3$. Then assumption (A) holds.

Proof. For $z \in \mathbb{R}$, note that

\begin{align*} \left( \prod_{\substack{j = 1 \\ \mu_{j} + z \neq 0}}^p {z + \mu_j} \right)\left( \sum_{\substack{i = 1 \\ \mu_{i} + z\neq 0}}^p \frac{\lambda_i}{z + \mu_i} \right) = \sum_{\substack{i = 1 \\ \mu_{i} + z \neq 0}}^p \lambda _i \prod_{\substack{j = 1 \\ j \neq i \\ \mu_{j} + z \neq 0}}^p \left(z + \mu_j\right)\end{align*}

is a polynomial in *$z$* of degree at most *$p-1$*. Hence it can admit at most *$p-1$* distinct roots. Now since there are at least $p+3$ regions where each the $k^{i_s}_3$ and the $k^{i_s}_2 + k^{i_s}_3$ are pairwise distinct, for at least four of them, say $i_1,\ldots,i_4$, $z = k^{i_s}_2 + k^{i_s}_3$ cannot be a root of the above polynomial. Further, for those four regions, since the $k^{i_s}_3$ are pairwise distinct, for any given $\mu_{j_0}$, at most one can be such that $\mu_{j_0} + k_3^{i_s} = 0$. As a consequence, the remaining three are such that the conditions of assumption (A) hold. □

Based on assumption (A), we now obtain the following proposition, which is the technical basis for our subsequent results on unique identifiability.

Proposition 12. Let $(p,n,((\lambda_j,\mu_j))_{j = 1}^p,((K_1^i,k_2^i,k_3^i))_{i = 1}^n,(C^i_{\textrm{T}})_{i = 1}^n,C_{\textrm{P}})$ be a configuration of the irreversible two tissue compartment model with $\mu_j \neq 0$ for $j = 1,\ldots,p$, $p \unicode{x2A7E} 3$, $n \unicode{x2A7E} 3$, $K_1^i,k_2^i,k_3^i > 0$ for all $i = 1,\ldots,n$ and such that assumption (A) holds. Let further $t_1,\ldots,t_T$ be distinct points such that

\begin{align*} T \unicode{x2A7E} 2\left(p+3\right).\end{align*}

Then, with $(\tilde p,n,((\tilde \lambda_j,\tilde \mu_j))_{j = 1}^{\tilde p} ,((\tilde K_1^i,\tilde k_2^i,\tilde k_3^i))_{i = 1}^n,(\tilde C^i_{\textrm{T}})_{i = 1}^n,\tilde C_{\textrm{P}})$ any other configuration of the irreversible two tissue compartment model such that $\tilde{p}\unicode{x2A7D} p$, $\tilde k_3^i \neq 0$ and $\tilde k_2^i+\tilde k_3^i \neq 0$ for all $i = 1,\ldots,n$, it follows from $C_{\textrm{T}}(t_l) = \tilde C_{\textrm{T}}(t_l)$ for $l = 1,\ldots, T$ that

\begin{align*} \tilde k_2^i = k_2^i \ \textrm{and } \tilde k_3^i = k_3^i\end{align*}

for all $i = 1,\ldots,n$, that there exists a constant *$\zeta\neq 0$* such that

\begin{align*} K_1^i = \zeta \tilde K_1^i\end{align*}

for all $i = 1,\ldots,n$, that $p = \tilde{p}$ and that (up to re-indexing)

\begin{align*} \tilde \mu_j = \mu_j \ \textrm{and } \tilde \lambda_j = \zeta \lambda_j \ \textrm{for all }j = 1,\ldots,p.\end{align*}

Proof. Take $(p,n,((\lambda_j,\mu_j))_{j = 1}^p,((K_1^i,k_2^i,k_3^i))_{i = 1}^n,(C^i_{\textrm{T}})_{i = 1}^n,C_{\textrm{P}})$ and $(\tilde p,n,((\tilde \lambda_j,\tilde \mu_j))_{j = 1}^{\tilde p},$

$((\tilde K_1^i,\tilde k_2^i,\tilde k_3^i))_{i = 1}^n,(\tilde C^i_{\textrm{T}})_{i = 1}^n,\tilde C_{\textrm{P}})$ to be two configurations as stated in the proposition, such that in particular

\begin{equation*} C_{\textrm{T}}^i\left(t_l\right) = \tilde C_{\textrm{T}}^i\left(t_l\right)\end{equation*}

for $l = 1,\ldots,T$.

Now since $\mu_j \neq 0$ for all $j = 1,\ldots,p$, we obtain the following representation of $C_{\textrm{T}}^i$:

\begin{align*} C_{\textrm{T}}^i\left(t\right) &amp;= \frac{K_1^i}{k_2^i+k_3^i}\sum_{j = 1}^p\left( \frac{k_3^i}{\mu_j}+\frac{k_2^i}{k_2^i+k_3^i+\mu_j}\mathbf{1}_{\left\{k_2^i+k_3^i+\mu_j\neq 0\right\}}\right)\lambda_je^{\mu_jt}\nonumber \\&amp;\quad -\left[\frac{K_1^ik_2^i}{k_2^i+k_3^i}\sum_{\substack{j = 1 \\ k_2^i+k_3^i+\mu_j\neq 0}}^p\frac{\lambda_j}{k_2^i+k_3^i+\mu_j}\right]e^{-\left(k_2^i+k_3^i\right)t} -\frac{K_1^ik_3^i}{k_2^i+k_3^i}\sum_{j = 1}^p \frac{\lambda_j}{\mu_j} \nonumber\\ &amp;\quad +\left[\frac{K_1^ik_2^i}{k_2^i+k_3^i}\sum_{\substack{j = 1 \\ k_2^i+k_3^i+\mu_j = 0}}^p\lambda_j\right]te^{-\left(k_2^i+k_3^i\right)t}.\end{align*}

In particular, for any region $i \in \{1,\ldots,n\}$, the coefficients of $e^{\mu_j t}$ for $j = 1,\ldots,p$ in this representation are given as either

\begin{align*} \frac{K_1^{i}\lambda_{j}k_3^{i}}{\mu_{j}\left(k_2^{i}+k_3^{i}\right)} \neq 0\end{align*}

in case $k_2^{i}+k_3^{i}+\mu_{j} = 0$ or

\begin{align*} \frac{K_1^{i}\lambda_{j}\left(\mu_{j}+k_3^{i}\right)}{\mu_{j}\left(k_2^{i}+k_3^{i}+\mu_{j}\right)}\end{align*}

otherwise. The latter can only be zero if $\mu_j + k_3^{i_0} = 0$, which can happen for at most one $\hat{j}$ by the $(\mu_j)_j$ being pairwise distinct. Since $p\unicode{x2A7E} 3$ by assumption, this implies in particular that $C_{\textrm{T}}^i$ is a non-zero function for any $i$. As a consequence of (5), the condition $T\unicode{x2A7E} 2(p+3)$ and the unique interpolation result of lemma 9, this implies that $\tilde C_{\textrm{T}}^i$ is a non-zero function, such that in particular $\tilde K_1^i\neq 0$ for all $i\in \{1,\ldots,n\}$. Together with the assumption that $\tilde k _3^i \neq 0$ for all *$i$*, we also obtain that $\tilde \mu_j \neq 0$ for all *$j$*, since otherwise $\tilde C_{\textrm{T}}^i$ would have a non-zero coefficient of *$t$*. As a consequence, also $\tilde C_{\textrm{T}}^i$ admits a representation as in (6).

**Uniqueness of the exponents $(\mu_j)_{j = 1}^p$.** As first step, we now aim to show that $\tilde{p} = p$ (in particular $\tilde{\lambda}_j\neq 0$ for all $j$) and that (up to re-indexing) $\mu_j = \tilde{\mu}_j$ for all $j = 1,\ldots,p$.

We start with a region $i_0 \in \{1,\ldots,n\}$. In this region, as argued above, the coefficients of the $e^{\mu_j t}$ for $j = 1,\ldots,p$ can be zero for at most one $\hat{j}$. Since further at most one *$j_0$* can be such that $\mu_{j_0} = -(\tilde k_2^{i_0} + \tilde k_3^{i_0})$, the unique interpolation result of lemma 9 applied to $C_{\textrm{T}}^{i_0}$ and $\tilde C_{\textrm{T}}^{i_0}$ yields that $\tilde{p}\unicode{x2A7E} p-2 \unicode{x2A7E} 1$ and that (up to re-indexing) $\mu_j = \tilde{\mu}_j$ for all $j \notin \{\hat{j},j_0\}$.

Now as a consequence of assumption (A), we can pick a region $i_1\neq i_0$ with $k_3^{i_1} \neq k_3^{i_0}$ where $\mu_{j_0} + k_3^{i_1}\neq 0$. Since already $\mu_{\hat j} + k_3^{i_0} = 0$ it further must hold that $\mu_{\hat j} + k_3^{i_1}\neq 0$. This means that the coefficients of both $e^{\mu_{\hat{j}}t}$ and $e^{\mu_{j_0}t}$ in the representation of $C_{\textrm{T}}^{i_1}$ as in (3) are non-zero. Again by the $\mu_j$ being pairwise distinct, this implies that $\tilde{p}\unicode{x2A7E} p-1$ and that (up to re-indexing) either $\mu_{\hat{j}} = \tilde \mu_{\hat{j}}$ or $\mu_{j_0} = \tilde \mu_{j_0}$.

**Case I.** Assume that $\mu_{\hat{j}} = \tilde \mu_{\hat{j}}$. If also $\mu_{j_0} = \tilde{\mu}_{j_0}$ (and $\tilde{p} \unicode{x2A7E} p$) we are done with this step, so assume the contrary.

Now as a consequence of assumption (A), we can pick $i_2,i_3$ and *$i_4$* to be regions where $\mu_{j_0} + k^{i_l}_3 \neq 0$ and either $\mu_{j_0} + k_2^{i_l} + k_3^{i_l} = 0$ or the coefficient of $e^{-(k_2^{i_l} + k_3^{i_l})t}$ in the representation of $C_{\textrm{T}}^{i_l}$ is non-zero. The fact that $\mu_{j_0} + k^{i_l}_3 \neq 0$, together with $\mu_{j_0} \neq \tilde{\mu}_{j_0}$ by assumption, further yields that $\mu_{j_0} = -(\tilde k_2^{i_l} + \tilde k_3^{i_l})$ for all $l = 2,3,4$.

Now we argue that in each region $i_l$ with $l = 2,3,4$, it must hold that either $-(k_2^{i_l} + k_3^{i_l}) = \tilde\mu_{j_0} $ or $k_2^{i_l} + k_3^{i_l} = \tilde k_2^{i_l} + \tilde k_3^{i_l}$. To this aim, we make another case distinction for a fixed $l \in \{2,3,4\}$.

**Case I. A** Assume that there exists *$j_l$* with $k_2^{i_l} + k_3^{i_l} + \mu_{j_l} = 0$. From the fact that this can happen at most for one $j_l$ and that $\lambda_{j_l} \neq 0$, it follows that the coefficient of $te^{-(k_2^{i_l} + k_3^{i_l})t}$ is non-zero. Consequently, it follows from the unique interpolation result that $k_2^{i_l} + k_3^{i_l} = \tilde k_2^{i_l} + \tilde k_3^{i_l}$ as claimed.

**Case I. B** Assume that $k_2^{i_l} + k_3^{i_l} + \mu_{j} \neq 0$ for all *$j$*. This means that $\tilde{\mu}_j = \mu_j \neq -(k_2^{i_l} + k_3^{i_l})$ for all $j\neq j_0$. But since the coefficient of $e^{-(k_2^{i_l} + k_3^{i_l})t}$ is non-zero, by the unique interpolation result it must hence hold that either $-(k_2^{i_l} + k_3^{i_l}) = \tilde\mu_{j_0} $ or $k_2^{i_l} + k_3^{i_l} = \tilde k_2^{i_l} + \tilde k_3^{i_l}$ as claimed. This concludes Case I.B.

Now given that either $-(k_2^{i_l} + k_3^{i_l}) = \tilde\mu_{j_0} $ or $k_2^{i_l} + k_3^{i_l} = \tilde k_2^{i_l} + \tilde k_3^{i_l}$ for all $l = 2,3,4$, one of the two cases must happen at least twice. By uniqueness of the $k_2^{i_l} + k_3^{i_l} $ for $l = 2,3,4$, only $k_2^{i_l} + k_3^{i_l} = \tilde k_2^{i_l} + \tilde k_3^{i_l}$ can happen twice. On the other hand, since $\mu_{j_0} = -(\tilde k_2^{i_l} + \tilde k_3^{i_l})$ for all $l = 2,3,4$, this yields that at least two $k_2^{i_l}+k_3^{i_l}$ coincide, which is a contradiction. Hence Case I is complete.

**Case II.** Assume that $\mu_{j_0} = \tilde \mu_{j_0}$. In this case, interchanging the role of $\mu_{j_0}$ and $\mu_{\hat{j}}$, we can argue that $\mu_{\hat{j}} = \tilde \mu_{\hat{j}}$ exactly as in Case I.

**Uniqueness of at least three of the exponents $k_2^{i}+k_3^{i}$.** Let $i_0$ be any region such that either $\mu_{j_0} + k_2^{i_0}+ k_3^{i_0} = 0$ for some $j_0 \in \{1,\ldots,p\}$ (such that the coefficient of $t e^{-(k_2^{i_l} + k_3^{i_l})t}$ in the representation of $C_{\textrm{T}}^{i_0}$ is non-zero) or the coefficient of $e^{-(k_2^{i_0} + k_3^{i_0})t}$ in the representation of $C_{\textrm{T}}^{i_0}$ is non-zero, and note that, according to assumption (A), at least three such regions exist.

**Case I.** Assume that there exists $j_0 \in \{1,\ldots,p\}$ such that $k_2^{i_0} + k_3^{i_0} + \mu_{j_0} = 0$. This implies that the coefficient of $te^{-(k_2^{i_0} + k_3^{i_0})t}$ is non-zero and, consequently, already that $k_2^{i_0} + k_3^{i_0} = \tilde k_2^{i_0} + \tilde k_3^{i_0}$ by uniqueness of exponents.

**Case II.** Assume that $k_2^{i_0} + k_3^{i_0} + \mu_{j} \neq 0$ for all $j = 1,\ldots,p$. Now since then the coefficient of $e^{-(k_2^{i_0} + k_3^{i_0})t}$ is non-zero by assumption, $-(k_2^{i_0} + k_3^{i_0})$ must match some exponent in the representation of $\tilde C^{i_0}_{\textrm{T}}$. It cannot match any of the $\tilde{\mu}_j = \mu_j $ since $k_2^{i_0} + k_3^{i_0} + \mu_{j} \neq 0$ for all $j = 1,\ldots,p$, hence again $k_2^{i_0} + k_3^{i_0} = \tilde k_2^{i_0} + \tilde k_3^{i_0}$ follows.

**Uniqueness of at least three of the exponents $k_2^i,k_3^i$**. First note that for any $i \in \{1,\ldots,n\}$ where $\tilde k_2^{i} + \tilde k_3^{i} = k_2^{i} + k_3^{i} $, from the unique interpolation result, it follows that

\begin{equation*} K^{i}_1 \lambda_j\left(\mu_j + k^ {i}_3\right) = \tilde K^{i}_1 \tilde \lambda_j\left(\mu_j + \tilde k^ {i}_3\right)\end{equation*}

for all $j = 1,\ldots,p$. Indeed, in case $k_2^{i} + k_3^{i} + \mu_j = 0$, it follows from the coefficients of $t e^{-(k_2^{i} + k_3^{i})t}$ in $C_{\textrm{T}}^i$ and $\tilde C_{\textrm{T}}^i$ being equal that

\begin{align*} \frac{K_1^{i} k_2^{i}\lambda_j}{k_2^{i}+k_3^{i}} = \frac{\tilde K_1^{i} \tilde k_2^{i}\tilde \lambda_j}{k_2^{i}+k_3^{i}},\end{align*}

which implies that $K_1^{i}k_2^{i} \lambda _j = \tilde K_1^{i} \tilde k_2^{i} \tilde \lambda _j$ and, using that $k_2^{i} = -\mu_j - k_3^{i}$ and $\tilde{k}_2^{i} = -\mu_j - \tilde{k}_3^{i}$, further yields $K^{{i}}_1 \lambda_j(\mu_j + k^ {{i}}_3) = \tilde K^{{i}}_1 \tilde \lambda_j(\mu_j + \tilde k^ {{i}}_3) $ as claimed.

In the other case, the equality (7) follows directly from the coefficients of $e^{\mu_j t}$ in $C_{\textrm{T}}^i$ and $\tilde C_{\textrm{T}}^i$ being equal.

Now let *i*_0_ be any region where $\tilde k_2^{i_0} + \tilde k_3^{i_0} = k_2^{i_0} + k_3^{i_0} $, and for which we want to show that $k_2^{i_0} = \tilde k_2^{i_0} $ and $k_3^{i_0} = \tilde k_3^{i_0} $. Again we consider several cases.

**Case I.** Assume that there exists $j_0 \in \{1,\ldots,p\}$ such that $ \mu_{j_0} + k_3^{i_0} = 0$. In this case, it follows from (7) that also $\mu_{j_0} + \tilde k_3^{i_0} = 0$ (note that $\tilde \lambda_{j_0} \neq 0 $ and $\tilde K_1^{i_0} \neq 0$ since $\tilde{p} = p$), hence $k_3^{i_0} = \tilde k_3^{i_0}$ and, consequently, $k_2^{i_0} = \tilde k_2^{i_0}$ holds.

**Case II.** Assume that $ \mu_{j} + k_3^{i_0} \neq 0$ for all *$j$*. In this case, using assumption (A) and the previous step, we can select *$i_l$* to be a second region where again $\tilde k_2^{i_1} + \tilde k_3^{i_1} = k_2^{i_1} + k_3^{i_1} $ and such that the $k_3^{i_0} \neq k_3^{i_1}$. We have two cases.

**Case II. A** Assume that there exists $j_1 \in \{1,\ldots,p\}$ such that $ \mu_{j_1} + k_3^{i_1} = 0$. As in Case I above, this implies that $k_3^{i_1} = \tilde k_3^{i_1}$. Further, choosing two indices $j_2,j_3 \in \{1,\ldots,p\}$ such that $j_1,j_2,j_3$ are pairwise distinct, it follows that $ \mu_{j_2} + k_3^{i_1} \neq 0$ and $ \mu_{j_3} + k_3^{i_1} \neq 0$ by the *$\mu_j$* being different. Using (7) and $k_3^{i_1} = \tilde k_3^{i_1}$ this implies

\begin{align*} K_1^{i_1}\lambda_{j_1} = \tilde K_1^{i_1} \tilde \lambda_{j_1} \qquad K_1^{i_1}\lambda_{j_2} = \tilde K_1^{i_1} \tilde \lambda_{j_2}.\end{align*}

Using that the $ \tilde K^{i_1}_1, \tilde K^{i_2}_1$ cannot be zero, these two equations imply

\begin{align*} \frac{\tilde \lambda_{j_1}}{\lambda_{j_1}} = \frac{\tilde \lambda_{j_2}}{\lambda_{j_2}}.\end{align*}

Combining this with the equations (7) for $i = i_0$ and $j = j_2,j_3$ we obtain

\begin{align*} \frac{\mu_{j_3}+\tilde k_3^{i_0}}{\mu_{j_3}+k_3^{i_0}} = \frac{\mu_{j_2}+\tilde k_3^{i_0}}{\mu_{j_2}+k_3^{i_0}}.\end{align*}

Reformulating this equation and using that $\mu_{j_2} \neq \mu_{j_3}$ this implies that $k_3^{i_0} = \tilde k_3^{i_0}$ and, consequently, $k_2^{i_0} = \tilde k_2^{i_0}$ holds.

**Case II. B** Assume that $ \mu_{j} + k_3^{i_1} \neq 0$ for all *$j$*. Defining $\Lambda_j = \tilde{\lambda}_j /\lambda_j$, we then obtain from (7) for pairwise distinct $j_1,j_2,j_3\in\{1,\dots, p\}$ that

\begin{align*} \Lambda_{j_1} \frac{\mu_{j_1}+\tilde{k_3^{i_s}}}{\mu_{j_1}+k_3^{i_s}} = \Lambda_{j_2} \frac{\mu_{j_2}+\tilde{k_3^{i_s}}}{\mu_{j_2}+k_3^{i_s}} = \Lambda_{j_3} \frac{\mu_{j_3}+\tilde{k_3^{i_s}}}{\mu_{j_3}+k_3^{i_s}}.\end{align*}

For $s = 0,1$. From this, we conclude that

\begin{equation*} 0 = \frac{\mu_{j_r}+\tilde k_3^{i_0}}{\mu_{j_r}+ k_3^{i_0}}\frac{\mu_{j_s}+\tilde k_3^{i_1}}{\mu_{j_s}+ k_3^{i_1}} - \frac{\mu_{j_r}+\tilde k_3^{i_1}}{\mu_{j_r}+ k_3^{i_1}}\frac{\mu_{j_s}+\tilde k_3^{i_0}}{\mu_{j_s}+ k_3^{i_0}}\end{equation*}

for $r,s \in \{1,2,3\}$ with *$r\neq s$*.

Multiplying (8) with the denominator $(\mu_{j_r}+ k_3^{i_0})(\mu_{j_s}+ k_3^{i_1})(\mu_{j_r}+ k_3^{i_1})(\mu_{j_s}+ k_3^{i_0})$ and further dividing by $(\mu_{j_r} - \mu_{j_s})$ we obtain

\begin{align*} 0 &amp;= \mu_{j_r}\mu_{j_s}\left(\tilde k_3^{i_0}-\tilde k_3^{i_1} +k_3^{i_1}-k_3^{i_0}\right)+\left(\mu_{j_r}+\mu_{j_s}\right)\left(k_3^{i_1}\tilde k_3^{i_0 }-k_3^{i_0}\tilde k_3^{i_1}\right)\\ &amp;\quad +\left(k_3^{i_1}-k_3^{i_0}\right)\tilde k_3^{i_0}\tilde k_3^{i_1}+\left(\tilde k_3^{i_0}-\tilde k_3^{i_1}\right)k_3^{i_0} k_3^{i_1}\end{align*}

for $r,s \in \{1,2,3\}$ with *$r\neq s$*. Subtracting the above equation for $(r,s) = (1,3)$ from the same equation for $(r,s) = (1,2)$ and dividing by $(\mu_{j_2} - \mu_{j_3})$ we obtain

\begin{equation*} 0 = \mu_{j_1}\left(\tilde k_3^{i_0}-\tilde k_3^{i_1}+k_3^{i_1}-k_3^{i_0}\right)+\left(k_3^{i_1}\tilde k_3^{i_0}-k_3^{i_0}\tilde k_3^{i_1}\right).\end{equation*}

Similarly, subtracting the above equation for $(r,s) = (2,3)$ from the same equation for $(r,s) = (2,1)$ and dividing by $(\mu_{j_1} - \mu_{j_3})$ we obtain

\begin{equation*} 0 = \mu_{j_2}\left(\tilde k_3^{i_0}-\tilde k_3^{i_1}+k_3^{i_1}-k_3^{i_0}\right)+\left(k_3^{i_1}\tilde k_3^{i_0 }-k_3^{i_0}\tilde k_3^{i_1}\right).\end{equation*}

Combining the last two equations and using that $\mu_{j_1} \neq \mu_{j_2}$ we obtain

\begin{align*} \tilde k_3^{i_0}-k_3^{i_0} = \tilde k_3^{i_1} - k_3^{i_1},\end{align*}

i.e. $\tilde k_3^{i_0} = k_3^{i_0} + \epsilon $ and $\tilde k_3^{i_1} = k_3^{i_1} + \epsilon $ for $\epsilon\in \mathbb{R}$. Inserting this into (9) we obtain

\begin{align*} \epsilon\left(k_3^{i_1} - k_3^{i_0}\right) = 0\end{align*}

which, together with $k_3^{i_1} \neq k_3^{i_0}$, yields *$\epsilon=0$* and hence in particular $k_3^{i_0} = \tilde{k}_3^{i_0}$ as desired. Together with $k_2^{i_0} + k_3^{i_0} = \tilde{k}_2^{i_0} + \tilde{k}_3^{i_0}$ this yields that also $k_2^{i_0} = \tilde{k}_2^{i_0}$.

**Uniqueness of the remaining $k_2^i,k_3^i$ and of the $K_1^i$ up to a constant factor.** Take $i_0$ to be a region where $\tilde k_2^{i_0} = k_2^{i_0}$ (we know already that such a region exists). It then follows from (7) that

\begin{equation*} K^{i_0}_1 \lambda_j = \tilde K^{i_0}_1 \tilde \lambda_j.\end{equation*}

For $j = 1,\ldots,p$. Thus, with $\zeta : = K^{i_0}_1 / \tilde K^{i_0}_1\neq 0$, we have that $\tilde \lambda_j = \zeta \lambda_j$ for all *$j$*. We now aim to show that, for all $i \in \{1,\ldots,n\}$, $k_2^i = \tilde k_2^i$, $k_3^i = \tilde k_3^i$ and $K_1^i = \zeta \tilde K_1^i$.

Consider $i \in \{1,\ldots,n\}$ fixed. To simplify notation, we drop here the index *$i$*, e.g. we write $K_1 = K_1 ^i$, $k_2 = k_2^i$ and $k_3 = k_3^i$ and similar for $\tilde K_1, \tilde k_2, \tilde k_3$.

In case $k_2 + k_3 + \mu_{j_0} = 0$ for some $j_0$, we know already from the previous step that $k_2 = \tilde k_2$ and $k_3 = \tilde k_3$, such that, from equating coefficients in the representations of *$C_{\textrm{T}}$* and $\tilde C_{\textrm{T}}$, we get

\begin{align*} K_1 \lambda_j = \tilde K_1 \tilde \lambda_j = \tilde K_1 \zeta \lambda_j,\end{align*}

such that also $K_1 = \zeta \tilde K_1$ as desired.

In the other case that $k_2 + k_3 + \mu_j \neq 0$ for all $j$, we get from equating coefficients in the representations of *$C_{\textrm{T}}$* and $\tilde C_{\textrm{T}}$, using $\tilde \lambda_j = \zeta \lambda_j$, that

\begin{equation*} \frac{\tilde K_1\zeta \left(\mu_j +\tilde k_3\right)}{\tilde k_2 + \tilde k_3 + \mu_j} = \frac{K_1\left(\mu_j + k_3\right)}{k_2 + k_3 + \mu_j} : = z_j\end{equation*}

for $j = 1,\ldots,p$, where the *$z_j$* are pairwise distinct by the *$\mu_j$* being pairwise distinct. Now we show that, from (12), it follows that $\zeta \tilde K_1 = K_1$, $\tilde k_2 = k_2$ and $ \tilde k_3 = k_3$. For this, we again need to distinguish several cases.

**Case I.** $\tilde{k}_3+\mu_{j_0} = 0$ for at least one $j_0 \in \{1,2,3\}$. This implies that also $k_3 + \mu_{j_0} = 0$ and hence that $\tilde k_3 = k_3$. Considering $j_1,j_2 \in \{1,2,3\} \setminus \{j_0\}$ with $j_1 \neq j_2 $ it follows from the $\mu_j$ being pairwise distinct that $k_3 + \mu_{j_s} \neq 0$ for $s = 1,2$, which implies that also $\tilde k_3 + \mu_{j_s} \neq 0$ for $s = 1,2$ and, consequently, that

\begin{equation*} \zeta \tilde K_1 = \frac{z_{j_s}}{\mu_{j_s} + k_3}\tilde k_2 + \frac{z_{j_s}}{\mu_{j_s} + k_3}\left(\mu_{j_s}+k_3\right)\end{equation*}

for $s = 1,2$. Now if $\frac{z_{j_1}}{\mu_{j_1} + k_3} \neq \frac{z_{j_2}}{\mu_{j_2} + k_3}$, one may derive $\tilde k_2 = k_2$ by rearranging the terms in (13) for $s = 1, 2$. Hence, by inserting the obtained equalities $\tilde k_2 = k_2$ and $\tilde k_3 = k_3$ in (12) we further deduce $\zeta \tilde K_1 = K_1$. If, on the other hand $\frac{z_{j_1}}{\mu_{j_1} + k_3} = \frac{z_{j_2}}{\mu_{j_2} + k_3}$ we can plug in the definition of $z_{j_1}, z_{j_2}$ and obtain

\begin{align*} \frac{K_1}{k_2 + k_3 + \mu_{j_1}} = \frac{K_1}{k_2 + k_3 + \mu_{j_2}},\end{align*}

which yields $\mu_{j_1} = \mu_{j_2}$ and hence a contradiction.

**Case II.** $\tilde k_3 + \mu_j \neq 0$ for all $j = 1,2,3$. In this case we can reformulate (12) to obtain

\begin{equation*} \zeta \tilde K_1 = z_j \frac{\tilde k_2 + \tilde k_3 + \mu_j}{\mu_j +\tilde k_3}\end{equation*}

for all $j = 1,2,3$. In particular, this yields

\begin{align*} z_1 \frac{\tilde k_2 + \tilde k_3 + \mu_1}{\mu_1 +\tilde k_3} = z_2 \frac{\tilde k_2 + \tilde k_3 + \mu_2}{\mu_2 +\tilde k_3}.\end{align*}

Now if $z_1(\mu_{2} + \tilde{k}_3) = z_2(\mu_{1} + \tilde{k}_3)$, this implies $\mu_1 = \mu_2$ and hence a contradiction. Thus, using that $z_1(\mu_{2} + \tilde{k}_3)\neq z_2(\mu_1 + \tilde{k}_3)$ we we can reformulate the previous equation to obtain

\begin{equation*} \tilde k_2 = \frac{\left(z_2-z_1\right)\left(\mu_2+\tilde k_3\right)\left(\mu_1+\tilde k_3\right)}{z_1\left(\mu_2+\tilde k_3\right) - z_2\left(\mu_1 + \tilde k_3\right)}.\end{equation*}

Now, in equation (12) for $j=3$, replacing $\zeta \tilde K_1$ by the equality (14) for $j=2$ and plugging in the expression (15) for $\tilde{k}_2$ we obtain, after some reformulations,

\begin{align*} &amp;\tilde k_3 \left[\left(z_3-z_2\right)\left(\mu_2-\mu_1\right)z_1 - \left(z_2-z_1\right)\left(\mu_3-\mu_2\right)z_3\right] \nonumber \\ &amp;\quad = \left(z_2-z_1\right)\mu_1\left[z_2\mu_3 - z_3 \mu_2\right] - \left(z_3-z_2\right)\mu_3\left[z_1\mu_2 - z_2 \mu_1\right].\end{align*}

Using the definition of the *$z_j$* in (12) we derive that the factor after $\tilde k_3$ in (16) corresponds to the term

\begin{align*} K_1^2 k_2 \frac{\left(\mu_2-\mu_1\right)\left(\mu_3-\mu_2\right)\left(\mu_1-\mu_3\right)}{\left(k_2+k_3+\mu_1\right)\left(k_2+k_3+\mu_2\right)\left(k_2+k_3+\mu_3\right)}\neq 0\end{align*}

which is nonzero by the *$\mu_j$* being pairwise distinct. Thus, again plugging in the definition of the *$z_j$* in (12) and rearranging the terms in (16) yields $\tilde k_3 = k_3$ after some computations and, consequently, also $\tilde k_2 = k_2$ and $\zeta \tilde K_1 = K_1$ by the previous considerations.

As a consequence, the remaining $\zeta \tilde K_1^i,\tilde k_2^i,\tilde k_3^i$ considered in this final part of the proof are uniquely determined as $\zeta \tilde K_1^i = K_1^i, \tilde k_2^i = k_2^i$ and $\tilde k_3^i = k_3^i$ □

The previous result shows that, already under knowledge of $C_{\textrm{T}}^i(t_l)$ for $i = 1,\ldots,n$ and sufficiently many distinct time-points *$t_l$*, the coefficients $k_2^i,k_3^i$ and the coefficients $K_1^i$ can be determined uniquely and uniquely up to a constant, respectively. Considering the ODE system (S), it is clear that this result cannot be improved in the sense that the constant factor of $K_1^i$ cannot be determined without any knowledge of *$C_{\textrm{P}}$* (since one can always divide all $K_1^i$ by a constant and multiply *$C_{\textrm{P}}$* by the same constant).

In case one aims to determine all parameters of a given configuration uniquely, some additional measurements related to *$C_{\textrm{P}}$* are necessary. It is easy to see that a single, non-zero measurement of *$C_{\textrm{P}}$*, for instance, would suffice. Indeed, given the value of a ground truth $\hat{C}_{\textrm{P}} (\hat{s})\neq 0$ at some time-point $\hat{s}$, the equality $C_{\textrm{P}}(\hat{s}) = \hat{C}_{\textrm{P}} (\hat{s}) = \tilde C_{\textrm{P}}(\hat{s}) $ together with the result from proposition 12 immediately imply that $\zeta=1$ such that all parameters are uniquely defined.

In current practice, indeed measurements of *$C_{\textrm{P}}$* are obtained via an expensive blood-sample analysis, and used for parameter identification, see for instance Veronese *et al* (2013). As discussed in the introduction, however, in contrast to obtaining measurements of $C_{\textrm{P}}$ *,* it is much simpler to obtain measurements of the total concentration *$C_{\textrm{WB}}$*, where $C_{\textrm{P}} = f C_{\textrm{WB}}$ with the unknown function *$f$*.

As the following result shows, measurements of $C_{\textrm{WB}}$ only are indeed sufficient to uniquely identify all remaining parameters, provided that one has sufficiently many measurements in relation to a parametrization of *$f$*. To formulate this, we need a notion of parametrization of the function $f(t)$.

Definition 13 (parametrized function class for **$f(t)$**).

For any $q \in \mathbb{N}$, we say that a set of functions $F_q \subset \{f:\mathbb{R} \rightarrow \mathbb{R}\} $ is a degree-*$q$* parametrized set if for any $f,\tilde{f}\in F_q$ and $\lambda \in \mathbb{R}$ it holds that $\lambda f - \tilde{f}$ attaining zero at *$q$* distinct points implies that *$\lambda=1$* and $f = \tilde{f}$.

Simple examples of degree-*$q$* parametrized sets of functions are polynomials of degree *$q-1$* that satisfy $f(x_0) = c$ for some given $x_0,c\in \mathbb{R} $ with $c\neq 0$ or polyexponential functions of degree $q/2$ (if *$q$* is even) that satisfy $f(x_0) = c$ for some given $x_0,c\in \mathbb{R} $ with *$c\neq 0$*. The latter is a frequently used type of parametrization for functions *$f(t)$* (where $f(0) = 1$ is required), see for instance Veronese *et al* (2013).

Proposition 14. In the situation of proposition 12, assume in addition that $f,\tilde{f}:\mathbb{R} \rightarrow \mathbb{R}$ are functions contained in the same degree-*$q$* parametrized set of functions, and are such that

\begin{align*} C_{\textrm{P}}\left(s_l\right) = f\left(s_l\right)C_{\textrm{WB}}\left(s_l\right) \ \textrm{and } \tilde C_{\textrm{P}}\left(s_l\right) = \tilde f\left(s_l\right)C_{\textrm{WB}}\left(s_l\right) \ \textrm{for }l = 1,\ldots,q,\end{align*}

with $s_1,s_2,\ldots,s_q$ being *$q$* different time points, and $C_{\textrm{WB}}(s_l)\neq 0$ given for $l = 1,\ldots,q$. Then, all assertions of proposition 12 hold with $\zeta=1$, and further

\begin{align*} f = \tilde{f}.\end{align*}

Proof. Proposition 12 already implies that $\tilde{C}_{\textrm{P}} = \zeta C_{\textrm{P}}$. Using that, by assumption,

\begin{align*} \zeta f\left(s_{l}\right)C_{\textrm{WB}}\left(s_{l}\right) = \zeta C_{\textrm{P}}\left(s_{l}\right) = \tilde C_{\textrm{P}}\left(s_{l}\right) = \tilde f\left(s_{l}\right)C_{\textrm{WB}}\left(s_{l}\right),\end{align*}

we obtain $(\zeta f - \tilde{f})(s_l) = 0$ for $l = 1,\ldots,q$. Since $f,\tilde{f}:\mathbb{R} \rightarrow \mathbb{R}$ are functions contained in the same degree-*$q$* parametrized set, this implies that $\zeta=1$ and $f = \tilde{f}$ as claimed. □

The following theorem now summarizes results of the previous two propositions in view of practical applications.

Theorem 15. Let $(p,n,((\lambda_j,\mu_j))_{j = 1}^p,((K_1^i,k_2^i,k_3^i))_{i = 1}^n,(C^i_{\textrm{T}})_{i = 1}^n,C_{\textrm{P}})$ be a ground-truth configuration of the irreversible two tissue compartment model such that

1. $p \unicode{x2A7E} 3$, $n\unicode{x2A7E} 3$ and $K_1^i,k_2^i,k_3^i > 0$ for all $i = 1,\ldots,n$,
2. There are at least *$p+3$* regions $i_1,\ldots,i_{p+3}$ where each the $k_3^{i_s}$ and the $k_2^{i_s} + k_3^{i_s}$ are pairwise distinct for $s = 1,\ldots,p+3$.

Let further $C_{\textrm{WB}}:[0,\infty) \rightarrow [0,\infty)$ be the ground truth arterial whole blood tracer concentration.

Then, for any other parameter configuration $(\tilde p,n,((\tilde \lambda_j,\tilde \mu_j))_{j = 1}^{\tilde p},((\tilde K_1^i,\tilde k_2^i,\tilde k_3^i))_{i = 1}^n,(\tilde C^i_{\textrm{T}})_{i = 1}^n$, $\tilde C_{\textrm{P}})$ such that the conditions (1) and (2) above also hold, it follows from

\begin{align*}C_{\textrm{T}}\left(t_l\right) = \tilde C_{\textrm{T}}\left(t_l\right) \quad \textrm{for }l = 1,\ldots, T\end{align*}

with $T \unicode{x2A7E} \max\{2(p+3),2(\tilde p + 3)\}$ and the $t_1,\ldots,t_T$ pairwise distinct, that, for some constant *$\zeta\neq 0$*,

\begin{align*} K_1^i = \zeta \tilde K_1^i, k_2^i = \tilde k_2^i \ \textrm{and } k_3^i = \tilde k_3^i\ \textrm{for all }i = 1,\ldots,n,\end{align*}

that $p = \tilde{p}$, and that (up to re-indexing)

\begin{align*} \tilde \mu_j = \mu_j \ \textrm{and } \tilde \lambda_j = \zeta \lambda_j \ \textrm{for all }i = 1,\ldots,p.\end{align*}

If further $f:[0,\infty) \rightarrow [0,\infty)$ is a ground-truth ratio between *$C_{\textrm{P}}$* and $C_{\textrm{WB}}$ in a degree-*$q$* parametrized set of functions and $\tilde f:[0,\infty) \rightarrow [0,\infty)$ is a function in the same degree-*$q$* parametrized set of functions such that

\begin{align*} C_{\textrm{P}}\left(s_l\right) = f\left(s_l\right)C_{{\textrm{WB}}}\left(s_l\right) \ \textrm{and } \tilde C_{\textrm{P}}\left(s_l\right) = \tilde f\left(s_l\right)C_{{\textrm{WB}}}\left(s_l\right) \ \textrm{for }l = 1,\ldots,q,\end{align*}

with the $s_1,\ldots,s_q$ pairwise distinct and $C_{{\textrm{WB}}}(s_l)\neq 0$ given, then *$\zeta=1$* and

\begin{align*} f = \tilde{f}.\end{align*}

Proof. This is an immediate consequence of lemma 11 and proposition 12: Indeed, lemma 11 ensures that the assumptions of proposition 12 are satisfied provided that (1.) and (2.) hold. In case $\tilde{p}\unicode{x2A7D} p$ the result immediately follows from propositions 12 and 14. In case $\tilde{p} > p$ it follows from interchanging the roles of the two configurations and again applying propositions 12 and 14. □

Remark 16 **(interpretation for practical application).**

Besides putting some basis assumptions on the ground truth-configuration and requiring positivity of the metabolic parameters, the previous theorem can be read as follows: If one obtains a configuration that matches the measured data, it can be guaranteed to coincide with ground-truth configuration if at least $\tilde{p}+3 $ of the found terms $\tilde k_2^i + \tilde k_3^i$ and $\tilde k_3^i$ are pairwise distinct.

Remark 17 **(generalization for nontrivial fractional blood volume).**

We assume here that the PET images provide exactly the tissue concentration *$C_{\textrm{T}}$*. A more realistic model would be that the voxel measurements provide a convex combination of the tissue and blood tracer concentration given by $C_{\textrm{PET}}(t) = (1-{V_{\textrm{B}}})\cdot C_{\textrm{T}}(t)+{V_{\textrm{B}}}\cdot C_{\textrm{WB}}(t)$, where ${V_{\textrm{B}}}$ with $0\unicode{x2A7D} {V_{\textrm{B}}}\unicode{x2A7D} 0.05$ describes the fractional blood volume. In case the parameter ${V_{\textrm{B}}}$ is known and *$C_{\textrm{WB}}$* is available at the same time points as the PET image measurements, our results cover also this setting. The general case, where both ${V_{\textrm{B}}}$ and *$C_{\textrm{WB}}$* are unavailable, can be addressed by similar techniques as in the proof of proposition 12. Here, the idea would be to employ a polyexponential parametrization also for *$C_{\textrm{WB}}$*, and assuming enough measurements of *$C_{\textrm{PET}}$* to be available in order to apply the unique interpolation result of lemma 9. One would further have to ensure positivity of *$C_{\textrm{WB}}$*, the initial condition $C_{\textrm{WB}}(0) = 0$ and conditions on the function $f = C_{\textrm{P}}/C_{\textrm{WB}}$ such as monotonicity and limiting conditions with respect to time approaching zero and infinity, respectively. These requirements imply corresponding conditions on the parameters of $C_{\textrm{P}}$ and *$C_{\textrm{WB}}$*.

## 4. A Tikhonov approach for parameter identification with noisy data

In the previous section we have established that, under appropriate conditions, the parameters $(K_1^i,k_2^i,k_3^i)$ of the irreversible two tissue compartment model in regions $i = 1,\ldots,n$ can be obtained uniquely from measurements $C_{\textrm{T}}(t_i)$, $i = 1,\ldots,T$ and measurements $C_{\textrm{WB}}(s_i)$ for $i = 1,\ldots,q$. While this result shows that parameter identification is possible in principle, it considers the idealized scenario of exact measurements.

In order to deal with noisy measurements, a standard technique in inverse problems is to employ a regularization approach and analyze (i) stability, i.e. if the proposed approach is stable with respect to (noise) variations in the measurements, and (ii) consistency, i.e. if, in the limit of vanishing noise, solutions of the approach converge to the ground truth parameters. In this section we consider Tikhonov regularization as a regularization approach and show that, with this, both stability (theorem 19) and consistency (theorem 21) can be obtained for our application at hand.

As first step, we define a forward model that maps the unknown parameters to the available measurement data. To this aim, we define the arterial concentration as mapping

\begin{equation*} \begin{aligned} C_{\textrm{P}}:~ \mathbb{R}^p\times\mathbb{R}^p &amp; \rightarrow \mathcal{P}_p \\ \left(\lambda, \mu\right) &amp; \mapsto \left[ t \mapsto \sum_{i = 1}^p \lambda_i e^{\mu_i t} \right]. \end{aligned}\end{equation*}

Further, we define a parametrized function as mapping

\begin{equation*} \begin{aligned} f: &amp; ~ \mathcal{M} \subset \mathbb{R}^{\hat{q}}\to F_q \\ &amp; ~ m\mapsto f_m, \end{aligned}\end{equation*}

where $\mathcal{M} \subset \mathbb{R}^{\hat{q}}$ is some (finite dimensional) parameter space and *$F_q$* is a degree-*$q$* parametrized set of functions.

Remark 18 **(*f*(*t*) example).**

A classical model for the function *$f(t)$* (see Tonietto *et al* (2015) for different models), that we will also use in our numerical experiments below, is the *biexponential model*

\begin{align*} f\left(t\right) = Ae^{\xi_1 t}+\left(1-A\right) e^{\xi_2 t} ~ ~ ~ \ \textrm{for } ~ ~ t\unicode{x2A7E} 0.\end{align*}

Here $\mathcal{M} = \left[0,\infty\right)\times\left(-\infty,0\right]^2$ and the degree of *$F_q$* is *$q=4$*.

In addition to the parameters of the functions modeling the arterial concentration and the parent plasma fraction, the forward model also includes the parameters $\textbf{K}^i = (K_1^i,k_2^i,k_3^i)$ for $i = 1,\ldots,n$ regions. With this, the unknown parameters are summarized by $(\lambda,\mu,m,\textbf{K}^i,\ldots,\textbf{K}^n)$ and we denote by $X = \mathbb{R}^p \times \mathbb{R}^p \times \mathbb{R}^{\hat q} \times \mathbb{R}^{3 \times n}$ the resulting parameter space with norm

\begin{align*} \Vert\left(\lambda, \mu, m, \textbf{K}^1, \dots, \textbf{K}^n\right)\Vert_X^2: = \sum_{j = 1}^p\left(\vert\lambda_j\vert^2+\vert\mu_j\vert^2\right)+\Vert m\Vert_2^2+\sum_{i = 1}^n\Vert\textbf{K}^i\Vert_2^2,\end{align*}

where $\|\cdot \|_2$ denotes the Euclidean norm. Given measurement points $t_1,\ldots,t_T$ for *$C_{\textrm{T}}$* and $s_1,\ldots,s_q$ for the total concentration *$C_{\textrm{WB}}$*, those parameters are mapped forward to a measurement space $Y = \mathbb{R}^{n\times T+q}$, again equipped with the Euclidean norm $\|\cdot \|_Y = \| \cdot \|_2$ via the function

\begin{equation*} \begin{aligned} F: \mathcal{D}\left(F\right): = \mathbb{R}^p\times\mathbb{R}^p \times \mathcal{M}\times \left[\epsilon, \infty\right)^{3\times n}\subseteq X &amp; \rightarrow Y \\ x &amp; \mapsto (F^1(x),F^2(x)) \end{aligned}\end{equation*}

where, for $x = (\lambda,\mu,m, \mathbf{K}^1, \dots, \mathbf{K}^n)$

\begin{equation*} F^1\left(x\right) = \begin{pmatrix} C_{\textrm{T}}\left(C_{\textrm{P}}\left(\lambda,\mu\right), \textbf{K}^1, \right)\left(t_1\right) &amp; \dots &amp; C_{\textrm{T}}\left(C_{\textrm{P}}\left(\lambda,\mu\right), \textbf{K}^1, \right)\left(t_T\right) \\ \vdots &amp; \ddots &amp; \vdots \\ C_{\textrm{T}}\left(C_{\textrm{P}}\left(\lambda,\mu\right), \textbf{K}^n, \right)\left(t_1\right) &amp; \dots &amp; C_{\textrm{T}}\left(C_{\textrm{P}}\left(\lambda,\mu\right), \textbf{K}^n, \right)\left(t_T\right) \end{pmatrix}\end{equation*}

and

\begin{equation*} F^2\left(x\right) = \begin{pmatrix} C_{\textrm{WB}}\left(s_1\right)f_m\left(s_1\right)-C_{\textrm{P}}\left(\lambda,\mu\right)\left(s_1\right) &amp; \dots &amp; C_{\textrm{WB}}\left(s_q\right)f_m\left(s_q\right)-C_{\textrm{P}}\left(\lambda,\mu\right)\left(s_q\right) \end{pmatrix}.\end{equation*}

Here $C_{\textrm{T}}\left(C_{\textrm{P}}\left(\lambda,\mu\right), \textbf{K}^i,\right)$ denotes the solution of the irreversible two tissue compartment ODE model (S) with parameters **$\textbf{K}^i$** and arterial concentration $C_{\textrm{P}}\left(\lambda,\mu\right)$. Note that *$F^2$* depends on the data *$C_{\textrm{WB}}$* that must be obtained from blood samples or PET measurements, which we assume to be given throughout this section. A further adaption of the model to include also *$C_{\textrm{WB}}$* as possibly noise measurement is possible with the same techniques as below, but will be omitted for the sake of simplicity.

Now denoting by $\hat{C}^i_{\textrm{T}}(t_1),\ldots,\hat{C}^i_{\textrm{T}}(t_T)$ for $i = 1,\ldots,n$ measurements corresponding to the ground-truth parameters, our goal is to find parameters $(\lambda,\mu,m,\textbf{K}^1,\ldots,\textbf{K}^n)$ such that

\begin{align*} F\left(\lambda,\mu,m,\textbf{K}^1,\ldots,\textbf{K}^n\right) = \begin{pmatrix} \hat C_{\textrm{T}}^1\left(t_1\right) &amp; \dots &amp; \hat C_{\textrm{T}}^1\left(t_T\right) \\ \vdots &amp; \ddots &amp; \vdots \\ \hat C_{\textrm{T}}^n\left(t_1\right) &amp; \dots &amp; \hat C_{\textrm{T}}^n\left(t_T\right) \end{pmatrix} \times \left(0,\ldots,0\right) \in \mathbb{R}^{n \times T} \times \mathbb{R}^q.\end{align*}

Accounting for the fact that the given parameters are perturbed by measurement noise, i.e. we are actually given $(C^i_{\textrm{T}})^\delta(t_l) $ with

\begin{align*} \sum_{i = 1}^n \sum_{l = 1}^T \|\left(C^i_{\textrm{T}}\right)^\delta\left(t_l\right) - \hat C^i_{\textrm{T}}\left(t_l\right) \|_2 ^2 \unicode{x2A7D} \delta,\end{align*}

we address the parameter identification problem via a minimization problem of the form

\begin{align*} \min_{\left(\lambda,\mu,m, \mathbf{K}^1, \dots, \mathbf{K}^n\right)\in \mathcal{D}\left(F\right)} \Vert F\left(\lambda,\mu,m, \mathbf{K}^1, \dots, \mathbf{K}^n\right)- \left(C_{\textrm{T}}^\delta,0\right)\Vert_Y^2 \nonumber\\ +\alpha\Vert \left(\lambda,\mu,m, \mathbf{K}^1, \dots, \mathbf{K}^n\right) - \left(\bar \lambda,\bar \mu,\bar m, \bar{\mathbf K}^1, \dots, \bar{\mathbf K}^n\right) \Vert_X^2.\end{align*}

Here $0 \in \mathbb{R}^q$ is a *$q$*-dimensional vector of zeros, $C_{\textrm{T}}^\delta$ summarizes the available measurements for $C_{\textrm{T}}^\delta$, i.e.

\begin{align*} C^\delta _{\textrm{T}} = \begin{pmatrix} \left(C_{\textrm{T}}^1\right)^\delta\left(t_1\right) &amp; \dots &amp; \left( C^1_{\textrm{T}}\right)^\delta\left(t_T\right) \\ \vdots &amp; \ddots &amp; \vdots \\ \left(C^n_{\textrm{T}}\right)^\delta\left(t_1\right) &amp; \dots &amp; \left(C_{\textrm{T}}^n\right)^\delta\left(t_T\right) \end{pmatrix}.\end{align*}

and $\left(\bar \lambda,\bar \mu,\bar m, \bar{\mathbf K}^1, \dots, \bar{\mathbf K}^n\right) $ is an initial guess on the ground truth parameters. The above approach corresponds to *Nonlinear Tikhonov-Regularization*, for which stability and consistency results can be ensured as follows.

Theorem 19 **(well-posedness and stability).**

Let the functions *$f_m$* be such that the mapping $m \mapsto f_m(t)$ is continuous for any $t \in [0,\infty) $. Then, for any given datum $C_{\textrm{T}}^\delta$, the minimization problem (21) admits a solution. Moreover, solutions are stable in the sense that, if $(C_{\textrm{T}}^{\delta_k})_k$ is a sequence of data converging to some datum $C_{\textrm{T}}^{\delta}$, then, any sequence of solutions $(x^k)_k$ of (21) with data $(C_{\textrm{T}}^{\delta_k})_k$ admits a convergent subsequence, and the limit of any convergent subsequence *$x$* is a solution of (21) with data $C_{\textrm{T}}^{\delta}$.

Proof. Since *$X$* and *$Y$* are finite dimensional and $\mathcal{D}(F)$ is obviously closed, this follows from classical results in regularization theory, see for instance Engl *et al* (2000, theorem 10.2) provided that $F$ is continuous.

We start with continuity of *$F^1$* as in (19), the first component of *$F$*. For this, it suffices to show that the mapping from the the parameter $(\lambda,\mu, \mathbf{K}^1, \dots, \mathbf{K}^n)$ to $C_{\textrm{T}}^i(t)$, with $t \in [0,\infty)$ fixed, is continuous, which, in turn, follows from the representation of $C_{\textrm{T}}^i(t)$ as in (1) if, for any $g \in L^2(0,t)$ and any sequence $(\lambda^l,\mu^l)_l$ converging to $(\lambda,\mu)$ it holds that

\begin{equation*} \int_0^tg\left(s\right)\left(C_{\textrm{P}}\left(\lambda^l,\mu^l\right)-C_{\textrm{P}}\left(\lambda,\mu\right)\right)\left(s\right)\mathop{}\!\mathrm{d} s \to 0 ~ ~ ~ \ \textrm{as } ~ ~ l\to\infty.\end{equation*}

By Hölder’s inequality, the latter follows from $C_{\textrm{P}}\left(\lambda^l,\mu^l\right)\to C_{\textrm{P}}\left(\lambda, \mu\right)$ in $L^2\left(0,T_{\textrm{max}}\right)$, which, in turn, follows via the Lebesgue dominated convergence theorem from point-wise convergence of $C_{\textrm{P}}\left(\lambda^l,\mu^l\right)$ and the fact that $|C_{\textrm{P}}\left(\lambda^l,\mu^l\right)|$ on $[0,t]$ can easily be bounded by a constant independent of *$l$*.

Regarding *$F^2$* as in (20), the second component of *$F$*, continuity immediately follows from continuity of $(\lambda,\mu) \mapsto C_{\textrm{P}}(\lambda,\mu)(t)$ and $m \mapsto f_m(t)$ for any $t \in [0,\infty)$ fixed, where the latter holds by assumption. □

Remark 20 (continuity of $m\mapsto f_m(t)$).

Note that the assumption of continuity of $m\mapsto f_m(t)$ is only necessary since we allow for arbitrarily parametrized functions *$f(t)$*; it holds in particular for the biexponential model of remark 18 and will typically hold for any reasonable parametrization.

At last in this section we now establish a consistency result.

Theorem 21 **(consistency).**

Let $(\hat p,n,((\hat \lambda_j,\hat \mu_j))_{j = 1}^{\hat p},((\hat K_1^i,\hat k_2^i,\hat k_3^i))_{i = 1}^n,(\hat C^i_{\textrm{T}})_{i = 1}^n,\hat C_{\textrm{P}})$ be a ground-truth configuration of the irreversible two tissue compartment model satisfying the assumptions of theorem 15, and let $f_{\hat m}\in \mathcal{M}$ be a ground-truth fraction.

With $\hat x = (\hat \lambda,\hat \mu,\hat m, \hat{\mathbf{ K}}^1, \dots, \hat{\mathbf{ K}}^n)$ the corresponding parameters and $\hat y : = F(\hat{x})$ the corresponding measurement data, let $y^{\delta_k}$ be any sequence of noisy data such that $\|\hat{y}-y^{\delta_k} \| \unicode{x2A7D} \delta_k$ with $\delta_k > 0$, $\lim_{k\rightarrow \infty} \delta_k = 0$.

Then, any sequence of solutions $(x_k)_k$ of (21) with data $y^\delta = y^{\delta_k}$ and $\alpha = \alpha_k$ such that $\alpha_k \rightarrow 0$ and $\delta_k^2/\alpha_k \rightarrow 0$ as $k \rightarrow 0$ admits a convergent subsequence. Any limit $ x = ( \lambda, \mu, m, \mathbf{ K}^1, \dots, \mathbf{K}^n)$ of such a subsequence, such that the corresponding parameter configuration satisfies the assumptions of theorem 15, coincides with $\hat{x}$. Further, if any limit of a convergent subsequence corresponds to a parameter configuration satisfying the assumptions of theorem 15, then the entire sequence $(x_k)_k$ converges to $\hat{x}$.

Proof. This is a consequence of theorem 15, which ensures that there is a unique $x \in X$ with $F(x) = \hat y$, and of classical results from regularization theory, see for instance Engl *et al* (2000, theorem 10.3). □

Remark 22 **(interpretation of the consistency result).**

When choosing $p\unicode{x2A7E} 3$ and $n\unicode{x2A7E} 3$, and given the definition of $\mathcal{D}(F)$ as in (18), the above consistency result together with the unique reconstructability result of theorem 15 can be interpreted as follows: Whenever the parameters $(K_1^i,k_2^i,k_3^i)_{i = 1}^n$ corresponding to a limit $x$ of $(x_k)_k$ are such that at least $p+3$ of the parameters $k^i_3$ and $k^i_2 + k_3^i$ are pairwise distinct, then one can ensure that $x = \hat{x}$.

Remark 23 **(multi-parameter regularization).**

The setting of (21) and the subsequent results on well-posedness and consistency can be generalized to incorporating different regularization parameters for the different norms and data terms, see for instance Holler *et al* (2018), which is reasonable given the fact that the parameters might live on different scales, and given the fact that the noise level of different measurements over time might be different.

Remark 24 **(model variations).**

Currently, in the setting of (21), the parameters $(K_1^i,k_2^i$, $k_3^i)_{i = 1}^n$ are bounded away from zero by *$\epsilon&gt;0$*. For the $(\mu_j)_{j = 1}^p$, we currently do not pose any constraints even though, as mentioned in remark 7, only the choice $\mu_j < 0$ is reasonable from a physiological perspective. Likewise, *$C_{\textrm{P}}$* as parametrized in (17), does not necessarily satisfy $C_{\textrm{P}}(0) = 0$. These two conditions, however, can be easily incorporated in the model via the additional constraint $\mu_j \unicode{x2A7D} -\tilde{\epsilon}$ for some $\tilde{\epsilon} \unicode{x2A7E} 0$ and via setting $\lambda_p = -\sum_{j = 1}^{p-1} \lambda _j$, respectively.

## 5. Numerical solution algorithm

The purpose of this section is to provide a proof-of-concept numerical setup that illustrates the analytic unique identifiability results of sections 3 and 4. Specifically, we consider the reconstruction of parameters from data simulated with the idealized forward model in case of noiseless and noisy measurements. The source code to reproduce all numerical experiments shown here can be found in Holler *et al* (2024).

We emphasize that, in line with the purpose of this section, important questions such as model error and uncertainty or applicability to real patient data are not considered here. Consequently, to confirm the practical feasibility of identifying metabolic parameters without the need of additional concentration measurements from blood samples, extensive further numerical experiments are necessary which are the scope of future work.

### 5.1. Simulation experiment setup

To evaluate our proposed kinetic parameter reconstruction method, realisitic time activity curves (TACs) obeying a realistic noise distribution and amplitude mimicking a dynamic [^18^F]-FDG brain acquisition were generated in the following way:

1. A ground truth arterial plasma concentation of the non-metabolized tracer was modeled by
  \begin{align*} C_{\textrm{P}}\left(t\right) = -10.9136 e^{-13.4522 t / \textrm{min}}+9.545 e^{-3.2672 t / \textrm{min}}+0.7331e^{-0.1532 t / \textrm{min}}+0.6355e^{-0.0106 t / \textrm{min}}\end{align*}
2. An ‘artificial’ ground truth ratio between arterial whole blood and non-metabolized plasma concentation was modeled by
  \begin{align*} f\left(t\right) = 0.2 e^{-0.2 t / \textrm{min}}+0.8e^{-0.005 t / \textrm{min}}\end{align*}
  which allows to calculate the ground truth total arterial whole blood tracer concentration using
  \begin{align*} C_{\textrm{WB}}\left(t\right) = C_{\textrm{P}}\left(t\right) / f\left(t\right).\end{align*}
  Note that this function is not representative for FDG, but was chosen to demonstrate the feasibility of the proposed approach.
3. Based on mean regional kinetic parameters $(K_1, k_2, k_3)$ reported for the control group in Jagust *et al* (1991),—shown in table 2—and equation (3), ground truth tissue activity concentrations $C_{\textrm{T}}^i(t)$ for the four regions frontal cortex, temporal cortex, occipital cortex and white matter were generated. Regional TACs were then calculated using $C_{\textrm{PET}}^i(t) = (1 - V_{\textrm{B}}) C_{\textrm{T}}^i(t) + V_{\textrm{B}} C_{\textrm{WB}}(t)$ and a fixed fractional blood volume of $V_{\textrm{B}} = 0.05$.
4. Ground truth dynamic activity image volumes were generated by assigning the values of $C_{\textrm{PET}}^i(t_k)$ to a sub-segmented version of the brainweb phantom mimicking an acquistion of $25$ dynamic time frames of $4\times5$ s, $4\times10$ s, $4\times30$ s, $2\times60$ s, $3\times150$ s, $6\times300$ s and $2\times600$ s post injetion. The values of $C_{\textrm{WB}}(t_k)$ were assigned to the vessel compartment of the brainweb phantom.
5. Noise-free TOF emission sinograms were generated from the dynamic PET image volumes using a realistic PET acqusition physics model of a state-of-the TOF PET scanner with an axial field of view of $20$ cm and TOF resolution of $400$ ps where the acquistion model included the effects of radioactive decay, photon attenuation, limited spatial resolution and a known additive contamination of random and scatter coincidences.
6. $20$ noise realizations of the emission sinogram at three different count levels (normal count, hight count, low count) were generated by adding Poisson noise to a scaled-version of the noise-free emission sinogram and reconstructed using OSEM ($6$ iterations, $28$ subsets). In the normal count scenario, the last $10$ min frame contained $70$ million true counts—which is representative for an acquisition using a $20$ cm axial FOV scanner and a injected dose of ca. $150$ MBq. The low and high count scenarios contained $10$ times less and more true coincidences, respectively.
7. 20 noisy regional tissue TACs $C_{\textrm{PET}}(t_k)$ were extracted by calculated the mean values in frontal cortex, temporal cortex, occipital cortex and white matter in all frames of the reconstructed dynamic PET image volumes. A noisy image-based estimate for $C_{\textrm{WB}}(t_k)$ was extracted by calculating the mean in the vessel ROI of the brainweb phantom.

**Figure 2.**
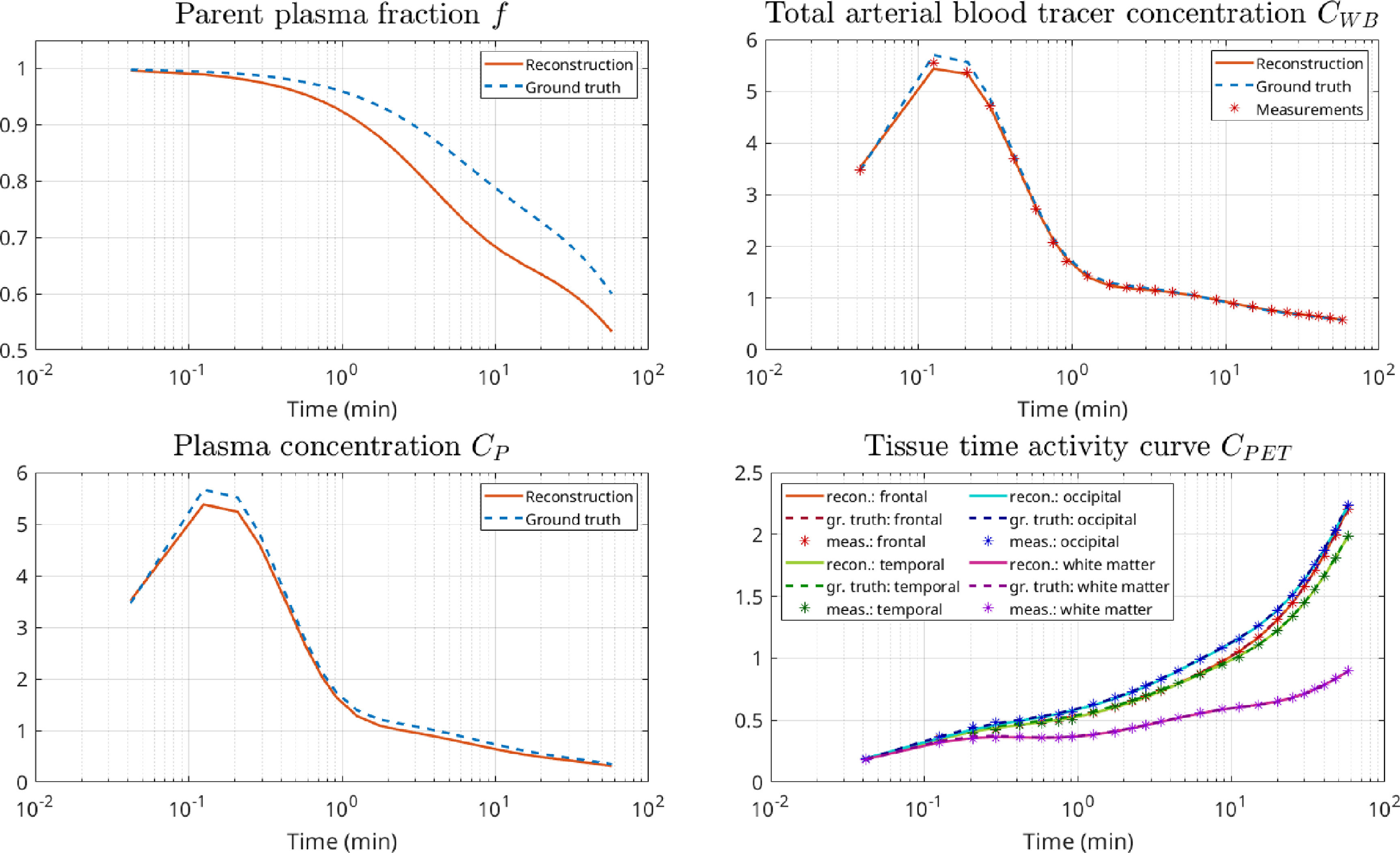
The subfigures display both the ground truth and exemplary reconstructed evolutions of the parent plasma fraction, total arterial blood tracer concentration, plasma concentration and tissue time activity curve. In case of *$C_{\textrm{WB}}$* and *$C_{\textrm{PET}}$* also the available noisy measurements are provided in the respective subfigures.

Figure 2 shows a visualisation of the function *$f(t)$*, the arterial plasma concentration, the total arterial whole blood tracer concentration and tissue time activity curve used for the simulation both for the ground truth curves and exemplary reconstructions that we will clarify later in more detail. Note that figure 2 is scaled logarithmically in time for a clearer visualization of the curvers at early times.

In view of propositions 12 and 14, the above experimental setting satisfies the assumptions such that unique identifiability from noiseless data can be guaranteed.

We summarize the unknown parameters of this setting by

\begin{align*} x^\dagger = \left(\lambda_1, \lambda_2, \lambda_3,\lambda_4, \mu_1, \mu_2, \mu_3,\mu_4, m_1, m_2, m_3, K_1^1, k_2^1, k_3^1, K_1^2, k_2^2, k_3^2, K_1^3, k_2^3, k_3^3, K_1^4, k_2^4, k_3^4\right)^T \in \mathbb{R}^{23},\end{align*}

where $x^\dagger$ denotes the ground-truth parameters as specified above. For a given number of measurements $T \in \mathbb{N}$, the data is summarized in vectorized form via

\begin{align*} y^\dagger = \left(y_1,0\right) = F\left(x^\dagger\right) \in \mathbb{R}^{4T+T},\end{align*}

where, abusing notation, $F:\mathbb{R}^{23} \to \mathbb{R}^{5T}$ is a vectorized version of the forward model of (18).

As we are dealing with locally convergent methods, it will also be important to choose a reasonable initial guess *$x_0$* for the algorithm. In order to test the performance of the algorithm in dependence on how close the initial guess is to the true solution, we employ the following steps to obtain perturbed initial guesses. Given a level of perturbation *$\delta_x$*, we define

\begin{equation*} x_0 = x^\dagger \left(1+\sigma \gamma\right)\end{equation*}

where $\sigma \sim \textrm{Unif}\left(\left\{-1,1\right\}\right)$, i.e. is uniformly distributed on $\{-1,1\}$ and $\gamma \sim \mathcal{N}\left(\delta_x,\frac{1}{4}\delta_x\right)$, i.e. is Gaussian distributed with mean *$\delta_x$* and variance $\delta_x/4$. This results in a expected squared deviation of *$x_0$* from $x^\dagger $ as by

\begin{equation*} \mathbb{E}\left(\frac{\Vert x_0-x^\dagger\Vert_X^2}{\Vert x^\dagger\Vert_X^2}\right) = \frac{1}{\Vert x^\dagger\Vert_X^2}\sum_{i = 1}^{18}\left(x^\dagger_i\right)^2\mathbb{E}\left(\sigma_i^2 \gamma_i^2 \right) = \mathbb{E}\left(\gamma_1^2\right) = \frac{1}{4}\delta_x+\delta_x^2,\end{equation*}

where $\sigma_i\sim \textrm{Unif}\left(\left\{-1,1\right\}\right) $ and $\gamma_i \sim \mathcal{N}\left(\delta_x,\frac{1}{4}\delta_x\right) $ for $i = 1,\ldots,23$ are independent random variables.

### 5.2. Algorithmic implementation

In order to numerically solve the non-linear parameter identification, we employ the *iteratively regularized Gauss–Newton method* of Bakushinskii (1992), see also Engl *et al* (2000, section 11.2). This is a standard method for solving non-linear inverse problems. It is related to the Tikhonov approach discussed in section 4 in the sense that similar results on stability and convergence/consistency (under appropriate source conditions) can be obtained, see for instance Blaschke *et al* (1994), Hohage (1997), but different to the Tikhonov approach, regularization is achieved by early stopping of the algorithm rather than adding an additional penalty term to the data-fidelity term. Early stopping has the advantage that, using an estimate of the noise level of the data, the discrepancy principle (Engl *et al* 2000, section 4.3) can be used to determine the appropriate amount of regularization, without requiring multiple solutions of a minimization problem as would be the case with the Tikhonov approach.

Given an initial guess $x_0 \in \mathcal{D}(F)$ and a sequence of regularization parameters $(\alpha_k)_k$ such that

\begin{equation*} \alpha_k&gt;0, ~ ~ 1\unicode{x2A7D} \frac{\alpha_k}{\alpha_{k+1}}\unicode{x2A7D} c_\alpha, ~ ~ \lim_{k\to\infty}\alpha_k = 0,\end{equation*}

where $c_\alpha > 1$ is some constant, the iteration steps of the iteratively regularized Gauss–Newton method for $k = 0,1,2,\ldots$ are given as

\begin{equation*} x_{k+1}^\delta = x_k^\delta+\left(F^{^{\prime}}\left[x_k^\delta\right]^TF^{^{\prime}}\left[x_k^\delta\right]+\alpha_kI\right)^{-1}\left(F^{^{\prime}}\left[x_k^\delta\right]^T\left(y^\delta-F\left(x_k^\delta\right)\right)+\alpha_k\left(x_0-x_k^\delta\right)\right),\end{equation*}

where $F^{^{\prime}}[x_k^\delta] \in \mathbb{R}^{(nT+q)\times (2p+3+2n)}$ denotes the Jacobian Matrix of *$F$* at $x_k^\delta$ and $F^{^{\prime}}\left[x_k^\delta\right]^T$ its transpose. Note that the approach is directly generalizable to incorporating different regularization parameters for the different parameter types by remark 23.

The iteration steps in (26) are repeated until the *discrepancy principle* is satisfied, that is, until $\Vert F\left(x_k^\delta\right)-y^\delta\Vert_Y\unicode{x2A7D} \tau\delta$ holds for the first time, where *$\delta$* is an estimate of $\|y^\delta - y^\dagger \|_Y$ and *$\tau&gt;1$* is a hyperparameter. The iterate *$x_k$* is then returned as the approximate solution of $F(x) \approx y^\delta$.

Remark 25 **(guaranteed convergence).**

Since the parameter identification problem addressed here is highly non-linear, global convergence guarantees for any numerical solution algorithm are out of reach. For the iteratively regularized Gauss–Newton method together with the discrepancy principle, as considered here, at least local convergence guarantees can be obtained as long as a particular source condition, i.e. a regularity condition on the ground truth solution holds, see Hohage (1997) for details.

In a practical application, the iteration (26) is combined with a projection on $\mathcal{D}(F)$, which is a closed, convex set for which the projection is explicit (we denote the projection map by $\mathcal{P}_{\mathcal{D}(F)}$), see Kaltenbacher and Neubauer (2006, theorem 4) for corresponding results on convergence of such a projected method. Together with this, we arrive at the algorithm for solving $F(x) \approx y^\delta$ as provided in algorithm 1, where we set $\epsilon = 10^{-3}$ for defining $\mathcal{D}(F) = \mathbb{R}^4 \times \mathbb{R}^4 \times [0,\infty) \times (-\infty,0]^2 \times [\epsilon,\infty)^{3\times 4}$. For the regularization parameters $\left(\alpha_i\right)_i$ we choose the ansatz

\begin{equation*} \alpha_i = a e^{-bi}\end{equation*}

for $i \in \mathbb{N}$ where $a\gg 1 $ and $0 < b < 1$ are fixed parameters. Besides fulfilling the decay conditions of (25), this choice is motivated by the goal of penalizing deviations from the initial guess rather strongly at early iterations (*$a$* large), and avoiding an exploding condition number of the matrix $\left(F^{^{\prime}}\left[x_k^\delta\right]^TF^{^{\prime}}\left[x_k^\delta\right]+\alpha_kI\right)$, that needs to be inverted at each iteration, during later iterations (*$b$* rather small).

**Table:** 

**Algorithm 1.** Parameter Identification by IRGNM.
**Input:** $\delta_x, \delta_y > 0, ~~ \tau > 1, ~~(\alpha_i)_i, ~~ x_0\in\mathcal{D}\left(F\right),~~ y^\delta \in Y, (C_{\textrm{WB}}(t_j))_{j = 1}^T$
**Initialise:** ${r_0} \leftarrow {y^\delta } - F\left( {{x_0}}\right),i\leftarrow 0$
**while** $\Vert r_i\Vert_Y > \tau\delta_y$ **do**
$\mathcal{A} \gets F^{^{\prime}}\left[x_i\right]^*$
$\mathcal{B} \gets \mathcal{A}\mathcal{A}^*$
*Solve* $~~\mathcal{B}\left(x-x_i\right) = \mathcal{A}r_i+\alpha_i\left(x_0-x_i\right)~~$ *for* $~~x\in X$
$x_{i+1} \gets \mathcal{P}_{\mathcal{D}\left(F\right)}\left(x\right)$
$r_{i+1} \gets y^\delta-F\left(x_{i+1}\right)$
$i\gets i+1$
**end while**
**return** *$x_k$*

For the realization of the forward operator *$F$* and the adjoint of its Fréchet-Differential the main idea is to vectorise the computations and omit expensive for-loops. For that, one may exploit that the entries of $F\left(x\right)$ and $F^{^{\prime}}\left[x\right]^*$, which mostly consist of integral type entities, may be computed analytically. The elementary components are of the form $\int e^{\mu s}\mathop{}\!\mathrm{d} s$, $ \int e^{\left(k_2+k_3\right)\left(s-t\right)}e^{\mu s}\mathop{}\!\mathrm{d} s$, $\int se^{\left(k_2+k_3\right)\left(s-t\right)}e^{\mu s}\mathop{}\!\mathrm{d} s$ and $\int se^{\mu s}\mathop{}\!\mathrm{d} s$. The latter may be computed by hand applying integration by parts. The corresponding terms, which depend on a combination of time evaluations, region and polyexponential parameters of *$C_{\textrm{P}}$*, are saved in three-dimensional tensors which are overloaded throughout a respective iteration to finally build up the adjoint operator of the Fréchet-Differential. For the implementation of the IRGNM we use the computational software Matlab (see MATLAB 2020).

## 6. Experimental results

The proposed method is tested for three different setups. The first is a reduced setup where we assume that the function *$f$* is known (i.e. the underlying parameters) and the measurements of $C_{\textrm{WB}}$ are noiseless. This corresponds to the situation that not only *$C_{\textrm{WB}}$*, but also measurements of the values of *$C_{\textrm{P}}$* are available at the time-points $t_1,\ldots,t_T$. While it is possible in practice to obtain those values via blood sample analysis, this procedure is time consuming and expensive, such that it is a relevant question to what extent such samples improve the identifiability of the tissue parameters. The second setup is a full setup where we assume that *$f$* is unknown and the measurements of *$C_{\textrm{WB}}$* are noiseless. Finally, the third setup is again a full setup but with noisy (e.g. image-based) measurements of *$C_{\textrm{WB}}$* based on the count setting. Note that in all three setups the tissue time activity curve *$C_{\textrm{PET}}$* is assumed to be available through noisy measurements with noise depending on the underlying count setting. In view of the data preprocessing it is important to note that bias correction techniques are not in the scope of this paper. Thus, the measurements of the total arterial blood tracer concentration *$C_{\textrm{WB}}$* are corrected for each time $t_1,\dots, t_T$ by a scaling factor which corresponds to the fraction of the ground truth *$C_{\textrm{WB}}$* by the mean of $20$ noisy realizations of *$C_{\textrm{WB}}$*. Similarly also the voxel measurements *$C_{\textrm{PET}}$* (see remark 17) are corrected and transformed to noisy realizations of the tracer concentration in tissue *$C_{\textrm{T}}$* for the different regions.

**Figure 3.**
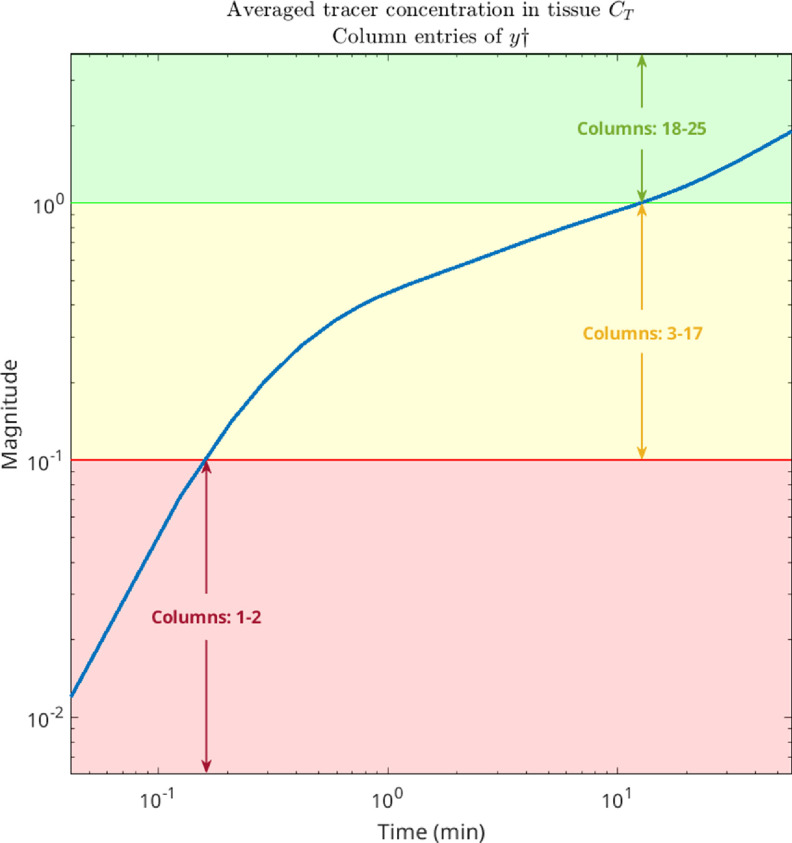
Visualization of magnitudes occurring in different time-intervals (i.e. columns) of the data $y^\dagger$.

In order to evaluate the identifiability of the parameters $x^\dagger$ from noisy data $y^\delta \approx F(x^\dagger)$, we consider both the situation of noiseless data and noisy data for the high, normal and low count setting. In case of noisy data, the discrepancy noise level $\delta_y > 0$ is estimated from the image data as follows. For a single noisy realization of measurements at fixed count setting we calculate at each time $t_1, \dots, t_T$ and for each region the fraction of the standard deviation of the tissue concentration in that region by its mean. Taking the mean of these fractions over the different times and regions together with the multiplication by the renormalization constant $1/400$ yields the discrepancy noise model. This results in $\delta_y\approx 0.003$ in the high count setting, $\delta_y\approx 0.011$ in the normal count setting and $\delta_y\approx 0.07$ in the low count setting. As can be observed in figure 3, which depicts the average magnitudes of different regions in the data, the noise level of the low count setting is already rather high in relation to the magnitude of the data.

For defining the initialization of the algorithm, we test with perturbations of the ground truth as defined in (23) for $\delta_x \in \{0.1, 0.2, 0.3, 0.4\}$. Recall that those are relative perturbations such that the square root of the expected squared deviation from the ground truth $x^\dagger$ is between ${\approx} 20\%$ for $\delta_x = 0.1$ and ${\approx} 50\%$ for $\delta_x = 0.4$.

To quantify the improvement compared to the initialization that is obtained by the algorithm, in addition to plotting the relative error $\frac{\|x_k - x^\dagger \|_X}{\|x^\dagger \|_X}$ over the iterations, we provide the following two values:

\begin{align*}\rho_\textrm{opt} = 100 \left( 1 - \Vert x_0^\delta -x^\dagger\Vert_X^{-1} \min_{1\unicode{x2A7D} k\unicode{x2A7D} \textrm{iter}_{\textrm{max}}} \Vert x_k^\delta -x^\dagger\Vert_X\right) \%\end{align*}

provides the best possible improvement (in percent, relative to the initialization) that was obtained during all iterations, and

\begin{align*} \rho_d = 100 \left( 1 - \Vert x_0^\delta -x^\dagger\Vert_X^{-1} \Vert x_N^\delta -x^\dagger\Vert_X\right) \%\end{align*}

provides the improvement that was obtained at iteration *N* where the algorithm was stopped by the discrepancy principle, i.e. the first iteration *N* where $ \Vert F\left(x_N^\delta\right)-y^\delta\Vert_Y\unicode{x2A7D} \tau \delta$ was fulfilled.

Simulations where carried out for each combination of different count settings and *$\delta_x$* as above. In order to obtain representative results, each experiment was carried out $20$ times. Those experiments where the algorithm diverged (i.e. no improvement compared to the initialization was achieved) were dropped (see table 1 for the number of dropped experiments using the kinetic parametes of the control group in Jagust *et al* (1991) for each parameter combination) and, among the remaining ones, the one whose performance was closest do the median performance of all repetitions was selected for the figures below.

**Table 1.** Number of experiments (out of $20$) not included in the final evaluation due to divergence using the kinetic parameters of the control group in Jagust *et al* (1991) (reduced setup/full setup with noiseless *$C_{\textrm{WB}}$*/full setup with noisy *$C_{\textrm{WB}}$*).

	${\delta_x = 0.4}$	${\delta_x = 0.3}$	${\delta_x = 0.2}$	${\delta_x = 0.1}$
noiseless	0/1/0	0/0/0	0/0/0	0/0/0
high count	0/4/2	0/5/3	0/2/0	0/3/0
normal count	2/5/3	2/7/3	2/4/0	2/5/0
low count	0/7/4	0/6/5	0/5/3	0/4/8

We recall that the hyperparameters of the chosen approach are the discrepancy principle parameter *$\tau$* in algorithm 1 and the regularisation parameters. As the ground truth parameters given in section 5.1 live on different scales we choose in view of remark 23 different regularization parameters for the metabolic parameters denoted by *$\alpha_i$*, the parameters of the plasma concentration *$C_{\textrm{P}}$* by *$\beta_i$* and those of the parent plasma fraction $f$ by *$\gamma_i$* in the form of the ansatz (27). This results in six different regularisation parameters (i.e. two for each parameter type). For the concrete hyperparameter tuning we proceed as follows for each setup introduced at the beginning of this section. We consider a different kinetic parameters taken from the AD group in Jagust *et al* (1991) for the normal count setting with fixed initial error level $\delta_x = 0.3$. For each combination of a large grid of different regularisation parameters we run the IRGNM for each of the $20$ realizations with disabled stopping criterion and calculate over $200$ performed iterations the optimal relative error of the metabolic parameters. From the resulting $20$ values we save the median. Finally the regularisation parameters are chosen such that the underlying combination of the grid minimizes the previously described median. With the tuned regularisation parameters at hand we similarly tune the discrepancy principle parameter *$\tau$*. Again for the dataset using the kinetic of the AD group in Jagust *et al* (1991) at normal count setting and $\delta_x = 0.3$ we run for a grid of *$\tau$*’s the IRGNM for each of the $20$ realizations with activated stopping criterion. Over the $200$ performed iterations, in case the stopping critertion applies, we calculate the relative error of the metabolic parameters at the iteration where the algorithm stops. In case a sufficient reduction is not achieved, i.e. the stopping criterion does not apply, we give the relative error of the metabolic parameters at the initial iteration. From the resulting $20$ values we save again the median and additionally the standard deviation. Finally, the discrepancy principle parameter *$\tau$* is chosen to minimize the previously described medians at a tradeoff of a small standard deviation which is important for a good generalization for unseen data. Note that tuning the hyperparameters using the AD group parameters and evaluating the results using the control group parameters of Jagust *et al* (1991) in the following, corresponds to the separation of data into a training and test set. Furthermore, by tuning the hyperparameters for the initial error level $\delta_x = 0.3$ at normal count setting it is expected that the method also generalizes well for slightly lower/higher *$\delta_x$* and count settings. For the reduced setup this results in the regularization parameters $\alpha_i = 10\cdot 2^{-i/5}$, $\beta_i = 600\cdot 2^{-i/7}$ and *$\tau=9.2$*. For the full setup with noiseless *$C_{\textrm{WB}}$* we derive $\alpha_i = 4000\cdot 2^{-i/7}, \beta_i = 100\cdot 2^{-i/7}, \gamma_i = 200\cdot 2^{-i/7}$ and *$\tau=6.8$*. For the full setup with noisy *$C_{\textrm{WB}}$* the hyperparameter tuning yields $\alpha_i = 3000\cdot 2^{-i/8}$, $\beta_i = 100\cdot2^{-i/8}$, $\gamma_i = 400\cdot 2^{-i/8}$ and *$\tau=17.6$*. While only results for the control group parameters (i.e. the test set) are shown below, the corresponding results for the AD group parameters (i.e. the training set) are shown in the supplementary material of this paper.

**Figure 4.**
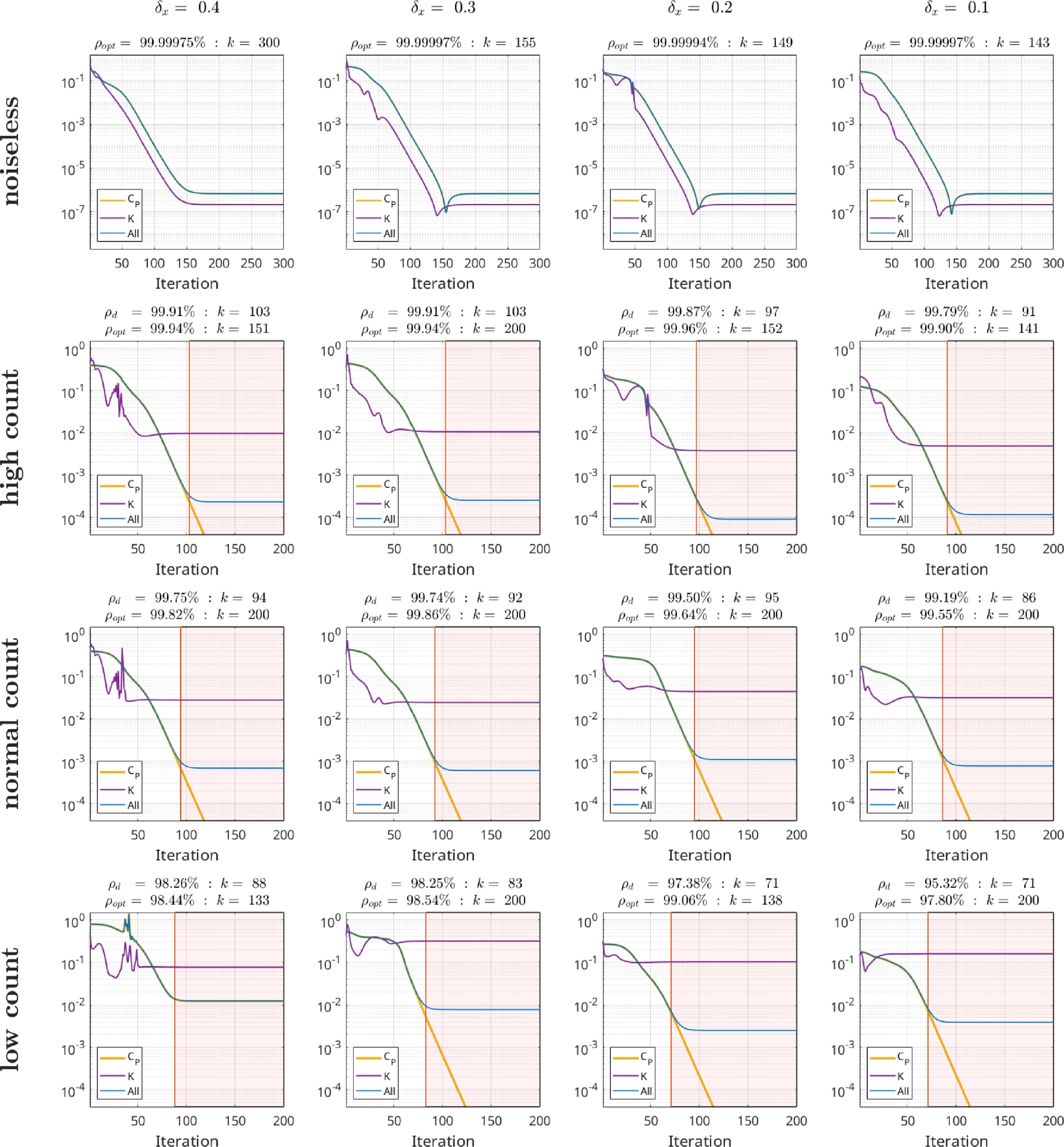
Performance of relative error $\Vert x^\dagger\Vert^{-1}_X\Vert x_k-x^\dagger\Vert_X$ under IRGNM for varying count settings (for measurements of tissue time activity curve *$C_{\textrm{PET}}$*) and different initial error levels in the reduced setup (i.e. for available function *$f$* and noiseless total arterial blood tracer concentration *$C_{\textrm{WB}}$*).

**Figure 5.**
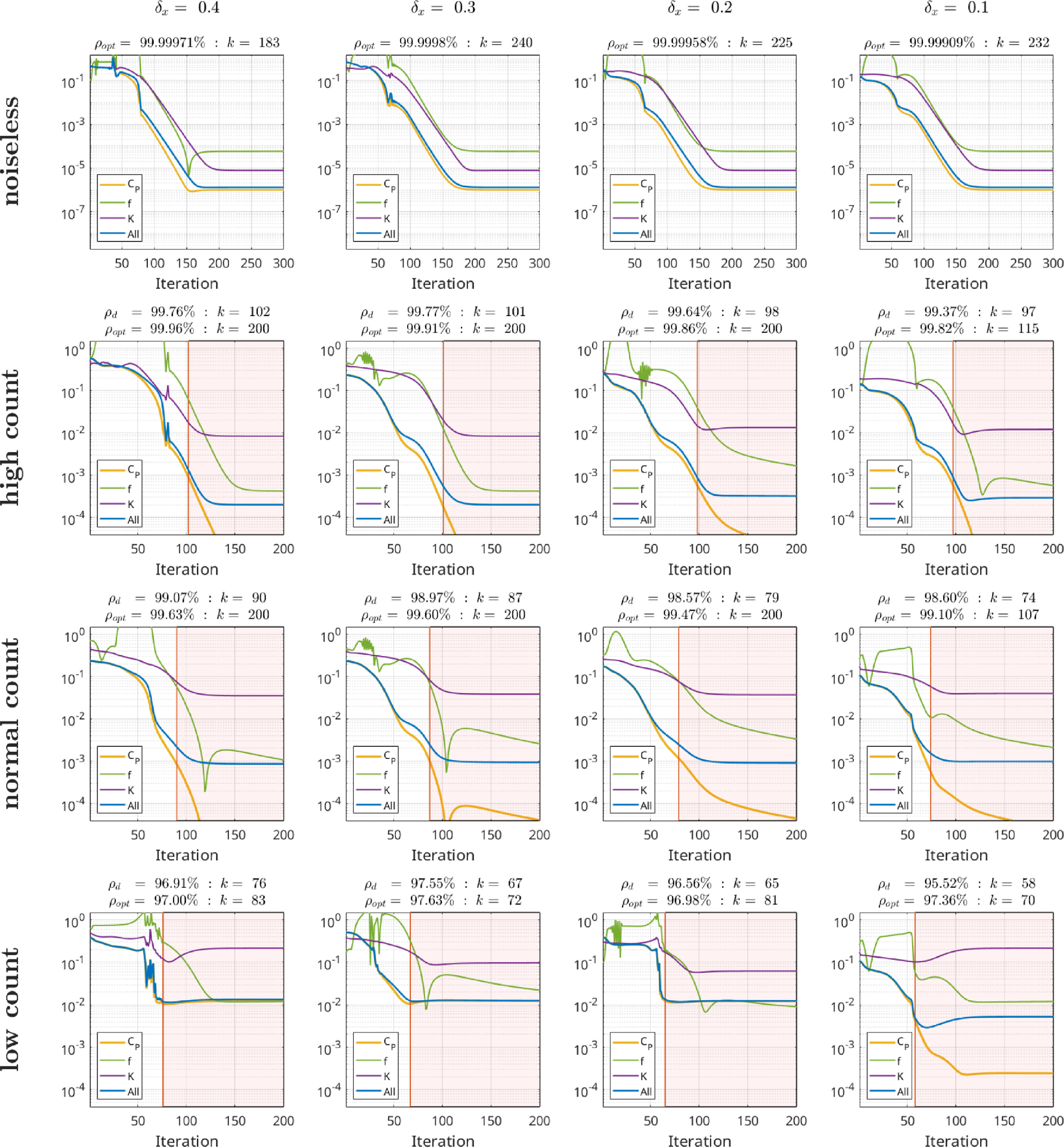
Performance of relative error $\Vert x^\dagger\Vert^{-1}_X\Vert x_k-x^\dagger\Vert_X$ under IRGNM for varying count settings (for measurements of tissue time activity curve *$C_{\textrm{PET}}$*) and different initial error levels in the full setup with noiseless *$C_{\textrm{WB}}$* (i.e. also function *$f$* is not available and total arterial blood tracer concentration *$C_{\textrm{WB}}$* is noiseless).

**Figure 6.**
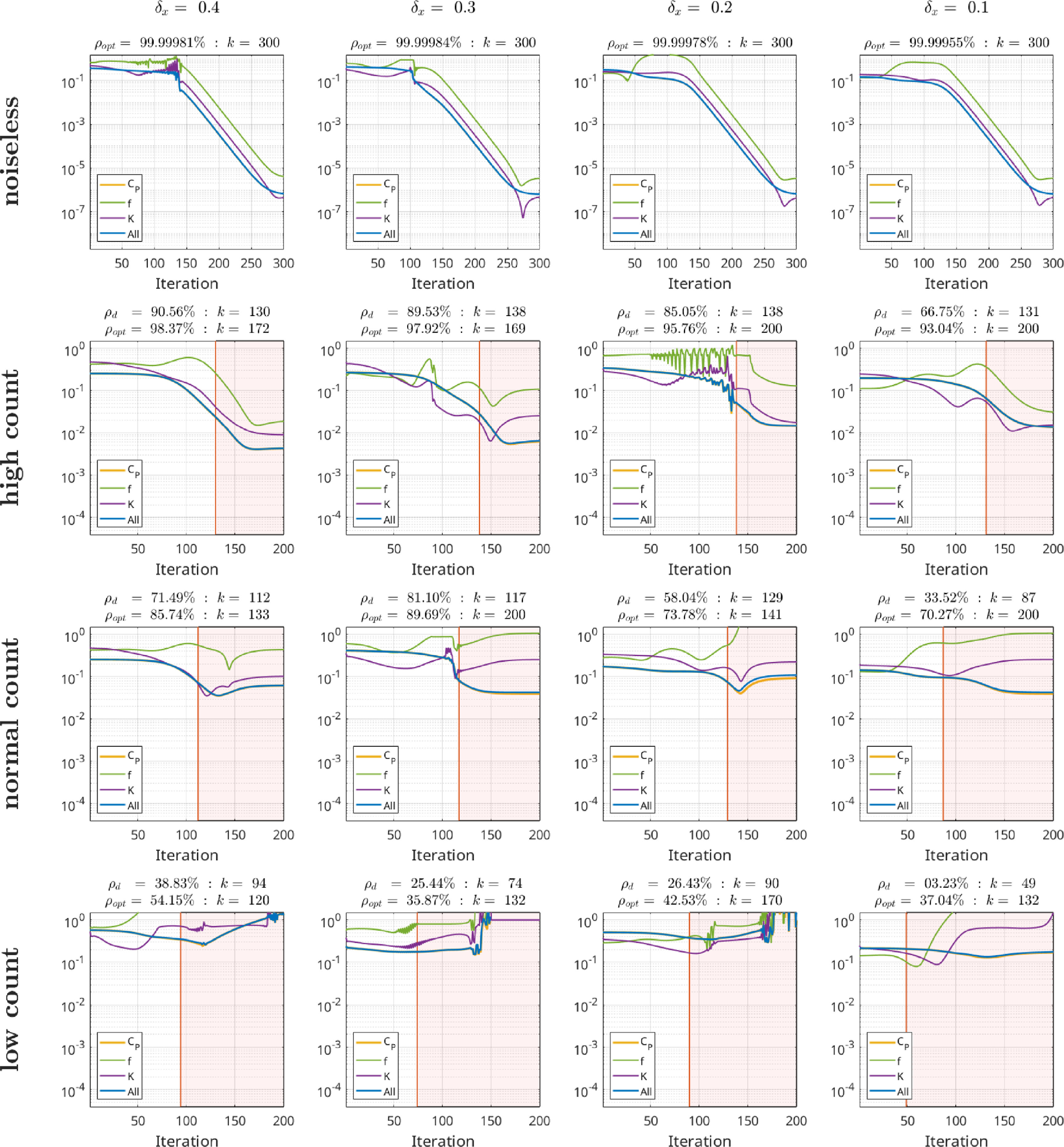
Performance of relative error $\Vert x^\dagger\Vert^{-1}_X\Vert x_k-x^\dagger\Vert_X$ under IRGNM for varying count settings (for measurements of tissue time activity curve *$C_{\textrm{PET}}$* and total arterial blood tracer concentration *$C_{\textrm{WB}}$*) and different initial error levels in the full setup with noisy *$C_{\textrm{WB}}$* (i.e. also function *$f$* is not available and total arterial blood tracer concentration *$C_{\textrm{WB}}$* is given by noisy measurements).

Figures 4–6 show the results obtained for the different setups introduced at the beginning of this section. They show the experiment whose performance was closest to the median performance (by discrepancy principle) of the non-divergent experiments for each combination of counting setups and initial error levels *$\delta_x$*. Recall that the number of divergent experiments out of $20$ realizations for each setup are shown in table 1. The reconstructions are considered both for the case of noiseless data and varying count settings in the respective subfigure rows. The subfigures depict the evolution of the relative error $\frac{\|x_k - x^\dagger \|_X}{\|x^\dagger \|_X}$ over iterations, respectively, with a logarithmic scale for the vertical axis. The different columns show results for the different choices of $\delta_x = 0.4,0.3,0.2,0.1$. The values of $\rho_\textrm{opt}$ and *$\rho_\textrm{d}$* (the latter only for $\delta_y > 0$), together with the respective iterations, are provided at the top of each subplot. Note that, while the algorithm was always run until a fixed, maximal number of iterations ($300$ for noiseless data and $200$ for the high/normal/low count setting) for obtaining the figures, in practice the iterations would be stopped by the discrepancy principle for $\delta_y > 0$. For $\delta_y > 0$, the red lines always indicate the iteration number where the algorithm would have stopped according to the discrepancy principle (i.e. when the the residual value $\Vert F(x_k)-y^\delta\Vert_Y$ subceeds the discrepancy level $\tau\delta_y$).

In the the first row of plots in figures 4–6, respectively, which considers the noiseless case, one can observe that the ground truth parameters $x^\dagger$ are approximated very well for all levels of *$\delta_x$*. This confirms our analytic unique identifiability result also in practice.

Considering figures 4–6, one can observe that, in the high counting regime, a good approximation of the ground truth $x^\dagger$ is still possible across different values of *$\delta_x$*. For higher noise, a good initialization (i.e. a small value of *$\delta_x$*) is of increasing importance. For the reduced setup depicted in figure 4 the reconstruction of the metabolic parameters starts becoming difficult for the low count setting. The same observation applies to the full setup with noiseless *$C_{\textrm{WB}}$* visualized in figure 5. For the result of the full setup with noisy *$C_{\textrm{WB}}$* given in figure 6 the reconstructions start to stagnate already at normal count setting and for the parameters of the parent plasma fraction *$f$*, the ground truth can not be recovered reasonably well. A reliable reconstruction for this setup is not possible in the low counting regime.

It can be observed that the performance with known *$C_{\textrm{P}}$* is improved compared to the situation where only *$C_{\textrm{WB}}$* is known, across all choices of counting settings and *$\delta_x$*. While for the noisless case and high count setting, both versions yield acceptable results, for the normal and low count setting knowledge of *$C_{\textrm{P}}$* enables a good approximation of the ground truth parameters in situations where this was not possible with knowing only *$C_{\textrm{WB}}$*. This indicates that, as one would expect, the problem of also identifying $f$ from measurements is significantly more difficult and there is a benefit (at least for this solution methods) in measuring *$C_{\textrm{P}}$* via blood samples. Moreover, there is obviously a benefit from knowing *$C_{\textrm{WB}}$* or *$C_{\textrm{WB}}$* being corrupted by less noise as can be seen by comparing figures 5 and 6.

Conclusively, we give the reconstructed metabolic parameters by the discrepancy principle for the setups introduced at the beginning of this section with normal count and initial error level $\delta_x = 0.3$ in the table 2 (compare to the relative error plots for normal count and $\delta_x = 0.3$ in figures 4–6). The metabolic parameters *$K_1, k_2$* and *$k_3$* are given for the different regions separately. Note further that the mean and standard deviation are calculated over the non divergent experiments out of $20$ given in table 1.

**Table 2.** Ground truth metabolic parameters and reconstructions for different setups. The value rec. is the reconstructed metabolic parameter value corresponding to the experiment depicted at subfigure row *normal count* and subfigure column $\mathit{\delta_x} = 0.3$ in the figures 4–6, respectively. Recall that the representative experiment depicted in each of the subfigures is the one whose relative error (with respect to all parameters including those of *$C_{\textrm{P}}$* and $f$) corresponds to the median over all non-divergent experiments, respectively. The value *mean* is the mean of reconstructed metabolic parameters over the non-divergent experiments whereas *std* the corresponding standard deviation.

	${K_1}$	${k_2}$	${k_3}$
	Ground truth parameters		
frontal	0.1570	0.1740	0.1180
temporal	0.1610	0.1790	0.0960
occipital	0.1770	0.1590	0.0880
white matter	0.1000	0.1610	0.0470
*rec. (mean/std)*	Reduced setup with noiseless *$C_{\textrm{WB}}$* at normal count and $\delta_x = 0.3$		
frontal	0.1579 (0.1571/0.0012)	0.1789 (0.1742/0.0046)	0.1202 (0.1180/0.0019)
temporal	0.1564 (0.1607/0.0039)	0.1676 (0.1783/0.0094)	0.0934 (0.0958/0.0019)
occipital	0.1733 (0.1764/0.0040)	0.1530 (0.1573/0.0098)	0.0872 (0.0873/0.0027)
white matter	0.0993 (0.0998/0.0012)	0.1584 (0.1603/0.0041)	0.0467 (0.0469/0.0007)
*rec. (mean/std)*	Full setup with noiseless *$C_{\textrm{WB}}$* at normal count and $\delta_x = 0.3$		
frontal	0.1562 (0.1582/0.0027)	0.1726 (0.1790/0.0128)	0.1174 (0.1227/0.0100)
temporal	0.1576 (0.1594/0.0051)	0.1718 (0.1751/0.0165)	0.0947 (0.0970/0.0066)
occipital	0.1841 (0.1765/0.0045)	0.1786 (0.1577/0.0121)	0.0930 (0.0895/0.0060)
white matter	0.0962 (0.1003/0.0026)	0.1450 (0.1619/0.0119)	0.0430 (0.0481/0.0025)
*rec. (mean/std)*	Full setup with noisy *$C_{\textrm{WB}}$* at normal count and $\delta_x = 0.3$		
frontal	0.1675 (0.1582/0.0057)	0.1718 (0.1694/0.0280)	0.1392 (0.1166/0.0148)
temporal	0.1623 (0.1618/0.0074)	0.1409 (0.1722/0.0252)	0.0955 (0.0937/0.0076)
occipital	0.1908 (0.1782/0.0089)	0.1589 (0.1537/0.0217)	0.1020 (0.0863/0.0077)
white matter	0.1036 (0.1008/0.0045)	0.1360 (0.1569/0.0199)	0.0469 (0.0460/0.0026)

**Figure 7.**
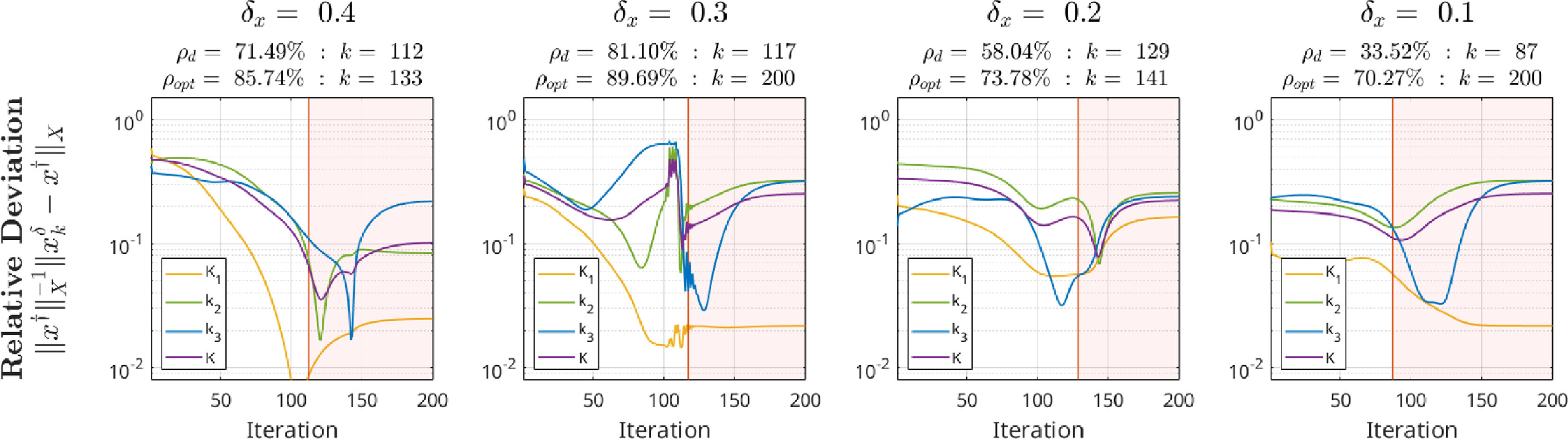
Relative error for different metabolic parameter types for full setup with noisy *$C_{\textrm{WB}}$* (i.e. function *$f$* is not available and total arterial blood tracer concentration $C_{\textrm{WB}}$ is given by noisy measurements), normal count (for measurements of tissue time activity curve *$C_{\textrm{PET}}$* and total arterial blood tracer concentration $C_{\textrm{WB}}$) and varying initial error level $\delta_x$.

Finally, in figure 7 we provide the relative error for the different metabolic parameter types separately for the full setup with noisy *$C_{\textrm{WB}}$* with normal count and $\delta_x = 0.3$. The results shown here suggests that the *$K_1$*-type parameter can be reconstructed better than *$k_2$* and *$k_3$*-type parameters.

## 7. Conclusions

In this work, we have shown analytically that most tissue parameters of the irreversible two-tissue compartment model in quantitative PET imaging can, in principle, be recovered from standard PET measurements only. Furthermore, a full recovery of all parameters is possible provided that sufficiently many measurements of the total arterial concentration are available. This is important, since it shows that parameter recovery is possible, at least in an idealized scenario, via using only quantities that are easily obtainable in practice, either directly from the acquired PET images or with a relatively simple analysis of blood samples.

While these results consider noiseless measurements, a connection to noisy measurements is made via showing that standard Tikhonov regularization applied to this setting yields a stable solution method that is capable of exact parameter identification in the vanishing noise limit.

These findings open the door to a comprehensive numerical investigation of parameter identification based on only PET measurements and estimates of the total arterial tracer concentration, using real measurement data and advanced numerical algorithms. While this paper contains a first numerical setup that illustrates its analytic results, a comprehensive effort, including important aspects such as model error, uncertainty and real-data experiments, is necessary to confirm our analytic results to be transferable to practice. This will be the next step of our research in that direction.

## Data Availability

All results of the paper can be reproduced using the source code publicly available in Holler *et al* (2024).
